# Cell morphology maintenance in *Bacillus subtilis* through balanced peptidoglycan synthesis and hydrolysis

**DOI:** 10.1038/s41598-020-74609-5

**Published:** 2020-10-21

**Authors:** Jad Sassine, Joana Sousa, Michael Lalk, Richard A. Daniel, Waldemar Vollmer

**Affiliations:** 1 Centre for Bacterial Cell Biology, Biosciences Institute, Faculty of Medical Sciences, Newcastle University, Newcastle upon Tyne, UK; 2 Institute of Biochemistry, University of Greifswald, 17489 Greifswald, Germany; 3 Present Address: Innovayt A/S, Avenida João Paulo II nº 30, 4715-034 Braga, Portugal; 4 Present Address: Department of Biochemistry, University of Oxford, Oxford, UK

**Keywords:** Microbiology, Bacteria

## Abstract

The peptidoglycan layer is responsible for maintaining bacterial cell shape and permitting cell division. Cell wall growth is facilitated by peptidoglycan synthases and hydrolases and is potentially modulated by components of the central carbon metabolism. In *Bacillus subtilis,* UgtP synthesises the glucolipid precursor for lipoteichoic acid and has been suggested to function as a metabolic sensor governing cell size. Here we show that *ugtP* mutant cells have increased levels of cell wall precursors and changes in their peptidoglycan that suggest elevated dl-endopeptidase activity. The additional deletion of *lytE*, encoding a dl-endopeptidase important for cell elongation, in the *ugtP* mutant background produced cells with severe shape defects. Interestingly, the *ugtP lytE* mutant recovered normal rod-shape by acquiring mutations that decreased the expression of the peptidoglycan synthase PBP1. Together our results suggest that cells lacking *ugtP* must re-adjust the balance between peptidoglycan synthesis and hydrolysis to maintain proper cell morphology.

## Introduction

The cell shape in most bacteria is maintained by the cell wall, which protects cells from bursting by the turgor^1^. *Bacillus subtilis* contains two major cell wall components, peptidoglycan (PG), and anionic polymers^2^. The mesh-like and stress-bearing PG is composed of glycan chains connected by short peptides and synthesized by glycosyltransferase (GTase) and transpeptidase (TPase) reactions^3,4^, which are provided by different classes of PG synthases, the Penicillin-binding proteins (class A and B), SEDS proteins and Mtg monofunctional GTases^2,5^. PBP1 is the most abundant bifunctional GTase–TPase active in vegetative *B. subtilis* cells, with roles in PG synthesis during both, length growth (cell elongation) and cell division^6–9^. Whilst cells lacking PBP1 are thinner^6^, the deletion of the other class A PBP genes encoding PBP2C, PBP4 and PBP2D has no major effect on cell morphology^10^.

The robust growth of the PG layer in *Escherichia coli* is likely facilitated by dynamic multi-protein complexes that contain PG synthases and hydrolases allowing the insertion of new material during cell elongation^11,12^. *B. subtilis* has at least 35 PG hydrolases with possibly redundant functions, which makes it difficult to unravel the physiological role of each enzyme^13–15^. The depletion of two dl-endopeptidases, CwlO and LytE causes the loss of rod-shape and eventually leads to cell death, hence, the activity of at least one of these enzymes is required to support PG growth and cell survival^16,17^. The ATP-binding cassette (ABC) transporter-like complex, FtsEX, interacts with and regulates the activity and localisation of CwlO along the cell periphery^18,19^. SweC and SweD form a complex with FtsX in the membrane and act as essential co-factors of FtsEX in controlling CwlO during cell elongation^20^. During vegetative growth LytE localises at mid-cell and in foci at the cell periphery and its absence results in bent cells and mild cell chaining suggesting a role in cell elongation and daughter cell separation^21–23^. The localisation of LytE at the cell periphery was diminished in an *mreBH* mutant and a genomic screen for MreBH interaction partners identified LytE, suggesting an interaction between the two proteins that may position LytE at the cell periphery prior to export to the cell wall^21^. Interestingly, the double mutants *mbl lytE* and *mreBH cwlO* are not viable suggesting that Mbl-CwlO (herein called CwlO system) and MreBH-LytE (LytE system) form two distinct cytoskeletal-endopeptidase systems of which the cell needs one for growth and survival^18,19^.

**Figure 1:**
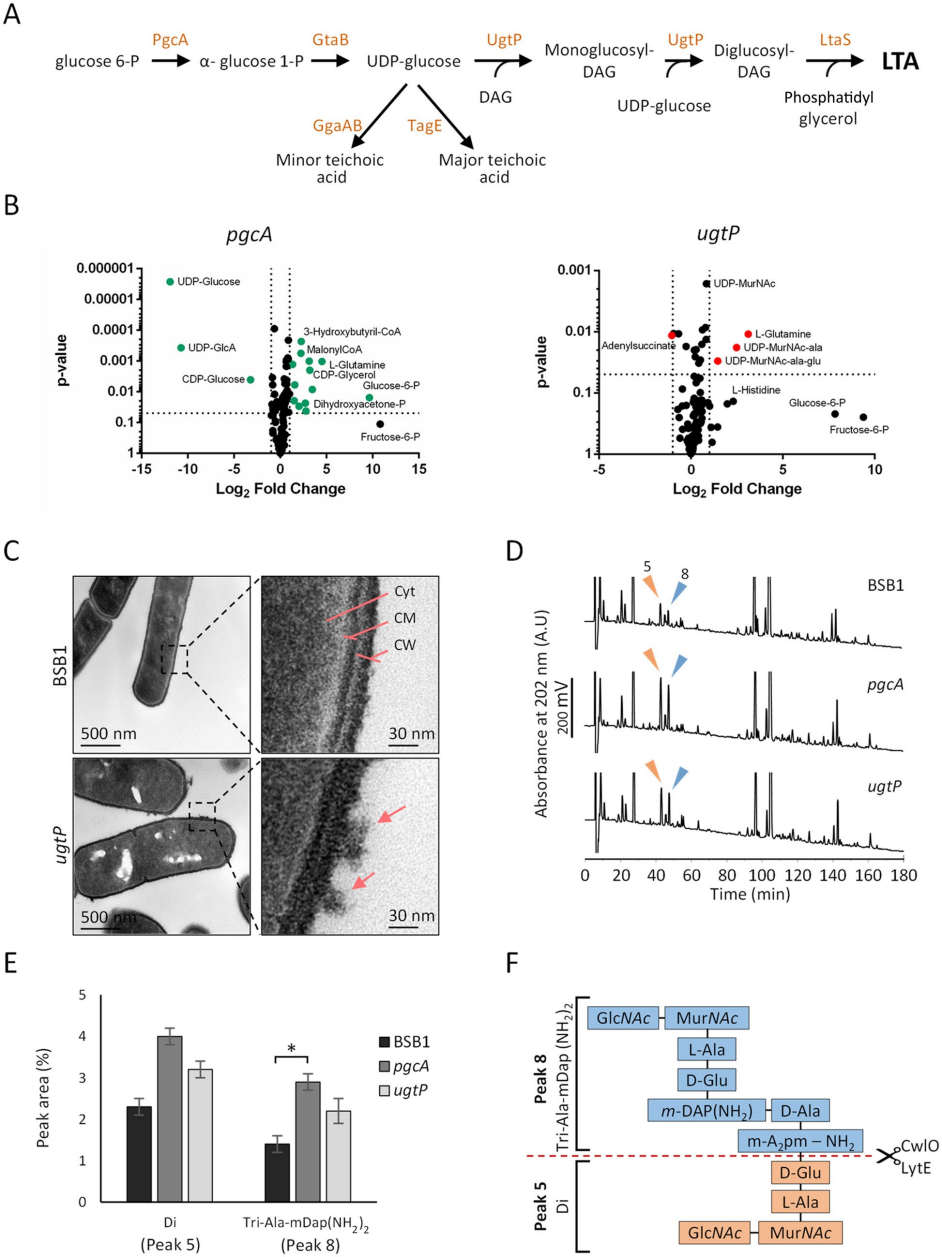
The *ugtP* mutant has altered cell surface and PG composition. (**A**) Teichoic acid glycolipids pathway of the cell wall in *B. subtilis*. DAG, diacylglycerol. (**B**) Volcano plot for data generated by metabolomics analysis for BSB1 Δ*ugtP* and BSB1 Δ*pgcA* showing increased levels of several lipid II precursors (**C**) Transmission electron microscopy (TEM) images showing a rougher cell surface structure in cells lacking UgtP compared to wild type cells. *Cyt* cytoplasm, *CM* cytoplasmic membrane, *CW* cell wall. Arrows indicate the rough cell surface. (**D**) HPLC analysis of muropeptides isolated from BSB1 Δ*ugtP* and BSB1 Δ*pgcA* mutants showed increased levels of Di (muropeptide 5) and Tri-Ala-mDap(NH_2_)_2_ (muropeptide 8) muropeptides compared to BSB1 suggesting increased endopeptidase activity. (**E**) Diagram representing the relative quantification of the two muropeptides in BSB1, BSB1 Δ*ugtP* and BSB1 Δ*pgcA*. *p ≤ 0.05. (**F**) The scheme represents the structure of the two muropeptides and the cleavage site of the LytE and CwlO endopeptidases.

Anionic polymers comprise wall teichoic acid (WTA), which is covalently bound to PG, and membrane-bound lipoteichoic acid (LTA)^2^. LTA has an important but as yet unclear role in controlling the activity of PG enzymes^24^, and an *ltaS* mutant lacking the major LTA synthase contained longer LTA polymers and increased expression levels of LytE^25–29^. LTA synthesis starts in the cytoplasm with the formation of glucose 1-phosphate and UDP-glucose by PgcA and GtaB, respectively (Fig. 1A). Subsequently, the glucosyltransferase UgtP transfers glucose residues to the membrane bound diacylglycerol moiety^30^, resulting in monoglucosyl diacylglycerol (MGDG), diglucosyl diacylglycerol (DGDG) and triglucosyl diacylglycerol (TGDG), forming 1.2%, 9.8% and 0.3% of the total membrane lipids^35^. The diglucosyl diacylglycerol and phosphatidyle glycerol are then utilised as substrates for LTA synthases (by LtaS, YfnI and YqgS) (Fig. 1A)^31–33^. However, *ugtP* mutant cells lack glucolipids but have LTA polymers suggesting that the addition of the two glucose residues to diacylglycerol is not required for LTA synthesis^34^. The loss of glucolipids has a pleiotropic effect on the cell and results in longer LTA molecules, swarming defect and increased stress response, however, the cause of these phenotypes remain unclear despite transcriptome analysis^36–38^.

In addition to its role in glucolipid and LTA synthesis, UgtP inhibits FtsZ assembly in a concentration dependent manner^39^. Alterations in nutrient availability causes variations in the level UDP-glucose which affects the expression and cellular localisation of UgtP. Hence, UgtP has been described as a metabolic sensor that couples Z-ring formation to growth rate^39,40^.

In this work we investigated how the loss of UgtP affects cell wall structure and synthesis. We discovered that in the absence of *ugtP,* PG synthesis and hydrolysis were both affected but the cell was capable of growing with normal rod-shape. The additional deletion of the hydrolase *lytE* resulted in sick, misshapen cells and these defects were alleviated by decreasing the expression of the synthase PBP1, thus allowing cells to regain the optimal balance between PG synthesis and hydrolysis. Deleting further genes to disturb this delicate balance in *ugtP* mutant cells allowed us to reveal the importance of a balanced PG synthesis and hydrolysis for rod-shape and, ultimately, cell survival.

## Results

### Deletion of *ugtP *or* pgcA* results in metabolic changes

Previous microarray experiments revealed that a *B. subtilis ugtP* deletion mutant showed altered levels of a large number of transcripts, which may explain the pleiotropic effects on cell growth and morphology associated with this strain^37^. However, the relative amount of mRNA in the cell does not always correlate with the level and activity of the corresponding gene products. Thus, to better understand the physiology of the *pgcA* or *ugtP* mutants we analysed the metabolome of cells grown to exponential phase in LB media and compared it to that of the parental strain BSB1. LC–MS and GC–MS identified 115 intracellular metabolites, including carbohydrates, organic acids, nucleotides, and amino acids and quantified their relative abundance (Supplementary Table S3). BSB1 Δ*ugtP* and BSB1 Δ*pgcA* showed changes in the levels of multiple metabolites compared to BSB1 and we highlight a few of the significant changes.

The loss of *pgcA* altered the relative levels of metabolites in the central carbon metabolism, especially the glycolysis pathway. The major differences were the notably increased level of glucose 6-phosphate (fold change = 796), followed by, dihydroxyacetone-phosphate (fold change = 11), 3-phosphoglycerate (fold change = 3), and other glycolytic intermediate metabolites with up to threefold in BSB1 Δ*pgcA* compared to BSB1 (Fig. 1B, Supplementary Table S3). As PgcA converts glucose 6-phosphate to glucose 1-phosphate the accumulation of its substrate, glucose 6-phosphate, was expected. This could also explain why fructose 6-phosphate and fructose 1,6-bis-phosphate (FBP) were present in higher levels whereas metabolites from the pentose shunt had similar levels in BSB1 Δ*pgcA* compared to BSB1. Later components of the glycolysis pathway were not seen to accumulate, consistent with a previous study showing the robustness of the metabolic network at the glucose 6-phosphate branch point in *B. subtilis*^41^.

Other perturbations were observed in the acetyl-CoA node with a significant increase in the levels of malonyl-CoA in BSB1 Δ*pgcA* compared to wild-type. The increased malonyl-CoA concentration in BSB1 Δ*pgcA* could reflect alterations of fatty acids synthesis which, in *B. subtilis*, is the only known cellular process that utilizes malonyl-CoA^42^. However, as fatty acids are essential components of the cellular membrane and a source for metabolic energy^43^, the implications of this are unclear.

Interestingly, the levels of several PG precursors increased in both, *pgcA* and *ugtP* mutants compared to BSB1, as seen when plotting the log_2_ Fold Change (log_2_FC) against the unpaired t-test results (volcano plot) (Fig. 1B, Supplementary Fig. S1). The main differences were observed for UDP-MurNAc-L-Ala, which increased 2.5-fold and 5.6-fold in the mutants lacking *pgcA* and *ugtP*, respectively (Supplementary Fig. S1, Supplementary Table S3). UDP-MurNAc-L-Ala-D-iGlu increased ~ 3-fold in both mutants compared to BSB1. The higher level of these intermediates could be due to an altered rate of PG precursor synthesis or disturbances in later stages of PG synthesis, either of which would potentially result in the accumulation of these precursors. PG precursors are synthesised by MurA, MurB and the amino acid ligases MurC-F, of which the expression of MurB and MurF are regulated by SigM. Transcriptomics analysis showed no significant changes in the mRNA levels of *mur* genes in the *ugtP* mutant compared to wild type cells^37^, however, the increased transcription of several sigma factors that mediate cell wall stress response, is consistent with higher Mur enzymes activity^38^.

### The ***ugtP*** mutant has an altered cell envelope structure

The increased level of PG precursors for BSB1 Δ*ugtP* together with the previously published increased sensitivity to high NaCl for strains lacking LTA glucolipids^44^ prompted us to further investigate the impact of the *ugtP* deletion on the ultrastructure of the cell envelope. Confirming a previous study^40^, we found that BSB1 Δ*ugtP,* BSB1 Δ*gtaB* and BSB1 Δ*pgcA* cells are shorter than cells of the wild-type (Supplementary Table S4), however, in the BSB1 background the loss of these genes did not result in a previously reported cell chaining morphology (Supplementary Fig. S2). To obtain high resolution images of the cell envelope, vegetative cells grown in LB were thin-sectioned and imaged by transmission electron microscopy (TEM). BSB1 Δ*ugtP* and BSB1 Δ*pgcA* (data not shown) was found to have rougher cell surface than wild type cells (Fig. 1C).

We next determined the muropeptide composition by reversed-phase HPLC after digestion with the muramidase cellosyl. The muropeptide profile of BSB1 was similar to that of the previously analysed *B. subtilis* wild type strain, 168CA^45,46^ (Fig. 1D, Supplementary Table S5). The profiles for BSB1 Δ*ugtP* and BSB1 Δ*pgcA* had the expected peaks previously observed for 168CA, however, the level of several crosslinked and non-crosslinked muropeptides were changed (Supplementary Table S5). Some of these changes were observed for the mono- or di-amidated muropeptides Tri(NH_2_) (peak 3), TetraTri(NH_2_) (peak 15) and TetraTri(NH_2_)_2_ (peak 21). Interestingly, BSB1 Δ*pgcA* and BSB1 Δ*ugtP* had increased levels of the muropeptides corresponding to disaccharide dipeptides (“Di”, peak 5) and a particular disaccharide pentapeptide (“Tri-Ala-mDap(NH_2_)_2_”, peak 8) (Fig. 1D,E). An increase of these muropeptides had previously been reported for strains with higher activity of the dl-endopeptidases CwlO or LytE (Fig. 1F)^16^. Thus, it is likely that one or more dl-endopeptidase(s) are upregulated or has a higher activity in cells lacking UgtP or PgcA.

### LytE is required to maintain rod-shape of the ***ugtP*** mutant

**Figure 2:**
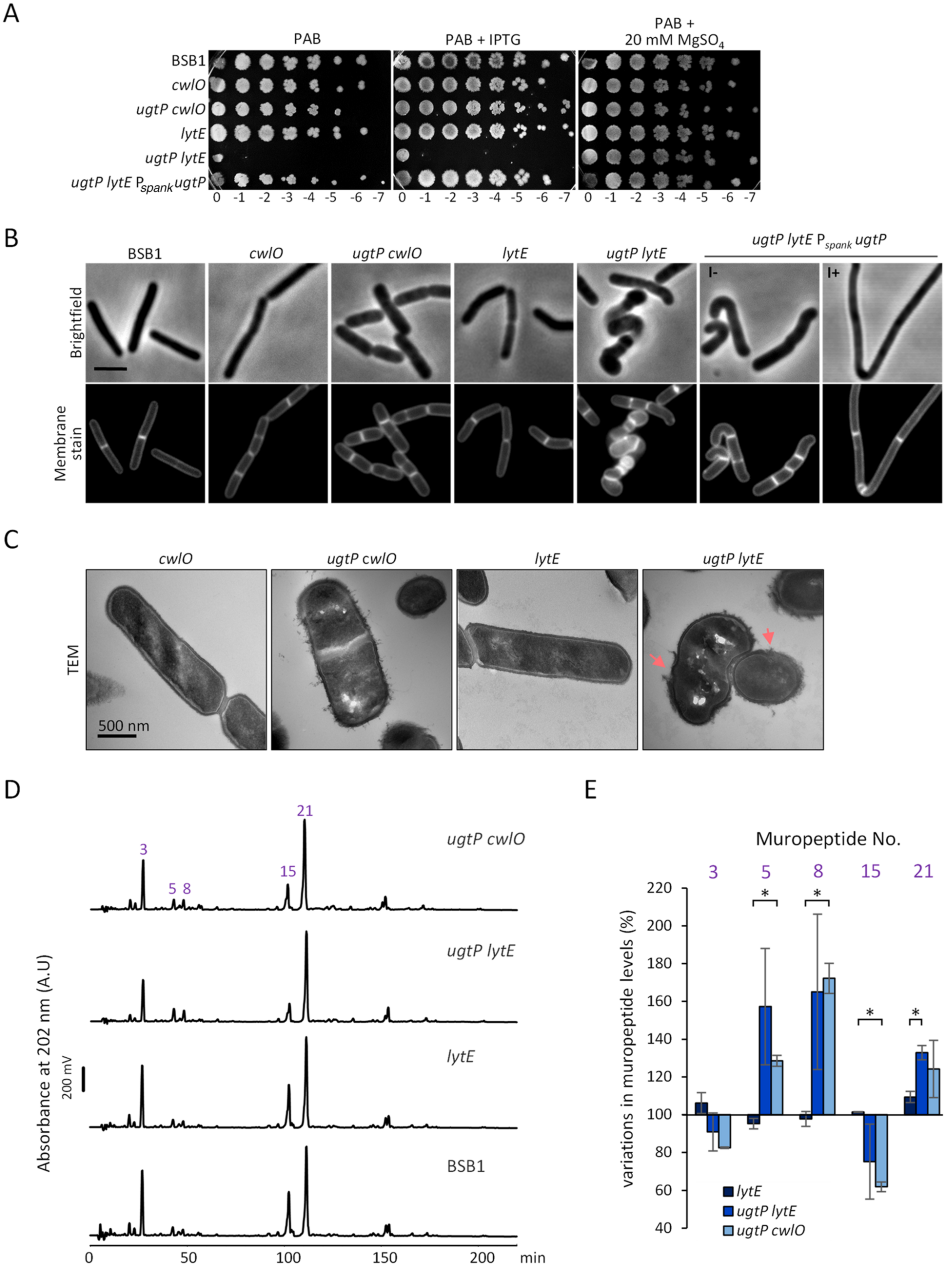
The *ugtP* mutant requires LytE to maintain rod-shape. (**A**) Spot plate assay showing the lethality of the *ugtP lytE* double knockouts when cells were grown on PAB media. The ectopic expression of UgtP or supplementing the plates with magnesium sulphate allowed BSB1 Δ*ugtP* Δ*lytE* to grow on PAB. (**B**) Fluorescence microscopy using brightfield and membrane stain to study the morphology of the mutants. BSB1 Δ*ugtP* Δ*lytE* showed severe shape defects during exponential phase when grown in LB media. The complementation of *ugtP* recovered this phenotype. *I−* without IPTG, *I+* with IPTG. Scale bar 3 µm. (**C**) TEM analysis of the mutants revealed the severity of the *ugtP lytE* morphology defects (arrows) where cells showed loss of rod-shape and membrane integrity. Arrows indicate ruptures in the cell wall (**D**) HPLC analysis of muropeptides isolated from BSB1 Δ*ugtP* Δ*lytE* and BSB1 Δ*ugtP* Δ*cwlO* showed alterations in the amidation patterns compared to BSB1. These alterations were mainly detected in the muropeptides Tri-(NH_2_) (muropeptide 3), TetraTri-(NH_2_) (muropeptide 15) and TetraTri-(NH_2_)_2_ (muropeptide 21). (**E**) Diagram representing the difference in the level of selected muropeptides in mutants compared to BSB1. 100% indicates the level of a muropeptide in BSB1. *p ≤ 0.05.

Cells lacking UgtP, PgcA or LtaS contain an increased level of LytE, but not CwlO, although the cellular localisation pattern of LytE is not affected^25^. To further study the importance of LytE and CwlO in the *ugtP* mutant, we constructed BSB1 Δ*ugtP* Δ*lytE* and BSB1 Δ*ugtP* Δ*cwlO,* and assessed their growth fitness in LB (not shown) and PAB media. Mutants showed aggravated phenotype when grown on PAB agar plates, presumably due to the presence of additional glucose, compared to LB, therefore, for clarity and consistency, spot plate assays were performed using PAB plates unless indicated otherwise. BSB1 Δ*cwlO*, BSB1 Δ*lytE* and BSB1 Δ*ugtP* Δ*cwlO* showed similar fitness as BSB1 (Fig. 2A, Supplementary Fig. S2). Interestingly, BSB1 Δ*ugtP* Δ*lytE* was not viable unless the PAB plates were supplemented with 20 mM magnesium sulphate. The expression of *ugtP* from the *amyE* locus allowed growth of the *ugtP lytE* strain showing that the absence of *ugtP* was responsible for the severe growth phenotype of the *ugtP lytE* mutant (Fig. 2A, Supplementary Fig. S2). We then determined cell morphology by light microscopy. Several of the mutants grew very poorly in PAB medium (data not shown), therefore, LB medium was used as an alternative in these experiments for consistency. BSB1 Δ*cwlO* cells were 16.6% shorter than wild type cells (Fig. 2B,C, Supplementary Table S4), consistent with previously published data^18^. BSB1 Δ*ugtP* Δ*cwlO* cells were 30% shorter than wild type cells. BSB1 Δ*lytE* had similar phenotype to BSB1 but occasionally produced bent cells whereas BSB1 Δ*ugtP* Δ*lytE* had severe shape defects, featuring bent cells and a loss of rod-shape (Supplementary Table S4). Similar differences in cell shape and growth defects were observed for BSB1 Δ*gtaB* Δ*lytE* compared to BSB1 Δ*gtaB* Δ*cwlO* cells (Supplementary Fig. S3). These results suggest that CwlO contributes less to the peripheral endopeptidase activity in the absence of *ugtP* and that the *ugtP* mutant relies on the activity of LytE to maintain rod-shape and viability.

The shape defects were confirmed by TEM which also showed a rough cell surface of the BSB1 Δ*ugtP* Δ*cwlO* mutant compared to BSB1 Δ*cwlO* and BSB1 Δ*lytE* mutants (Fig. 2C). In contrast, BSB1 Δ*ugtP* Δ*lytE* cells lost rod-shape and their cell envelope was occasionally ruptured, which is potentially related to the observed lysis in the absence of magnesium sulphate.

In an attempt to further understand the reasons behind the loss of rod-shape in BSB1 Δ*ugtP* Δ*lytE*, PG was isolated from exponentially growing cells and the muropeptides composition was compared to that of BSB1 Δ*lytE* and BSB1 Δ*ugtP cwlO*. Overall, the three mutant strains showed a muropeptide profile similar to BSB1 with the exception of minor changes in the levels of certain muropeptides (Fig. 2D, Supplementary Table S5). The PG from BSB1 Δ*ugtP* Δ*lytE* and BSB1 Δ*ugtP* Δ*cwlO* showed an increase in the Di and Tri-Ala-mDap-(NH_2_)_2_ muropeptides, comparable to the increase of these in BSB1 Δ*ugtP*, suggesting a higher dl-endopeptidase activity despite the absence of one of the two dl-endopeptidases (CwlO or LytE) (Fig. 2D,E, Supplementary Table S5). The increase of these muropeptides could indicate an activation/overproduction of one or more of the other dl-endopeptidases present in the double mutants^23,47^. Additionally, BSB1 Δ*ugtP* Δ*lytE* and BSB1 Δ*ugtP* Δ*cwlO* had a decrease, by up to 33%, in the levels of the mono-amidated muropeptides Tri-(NH_2_) and TetraTri-(NH_2_) compared to *lytE* and wild type cells. This decrease was paralleled by an increase in the level of the di-amidated muropeptide TetraTri-(NH_2_)_2,_ without affecting the total level of amidated muropeptides for all mutants (Fig. 2E, Supplementary Table S5). Similar changes in the amidated muropeptides were observed for BSB1 Δ*ugtP* and BSB1 Δ*pgcA*. *B. subtilis asnB* mutant, which is impaired in the amidation of PG, exhibited deregulated PG hydrolases^45,48^. Consistent with this data, several sick mutants in this study showed changes in the levels of amidated muropeptides, and improved growth and morphology in the presence of excess magnesium sulphate. Consequently, magnesium ions and amidation appear to help regulate PG synthesis and hydrolysis to maintain cell shape, however, more work is required to understand how these modifications affect the biosynthesis of the sacculus.

### Overexpression of CwlO ameliorates the growth and morphology of ***ugtP lytE*** mutant cells

**Figure 3:**
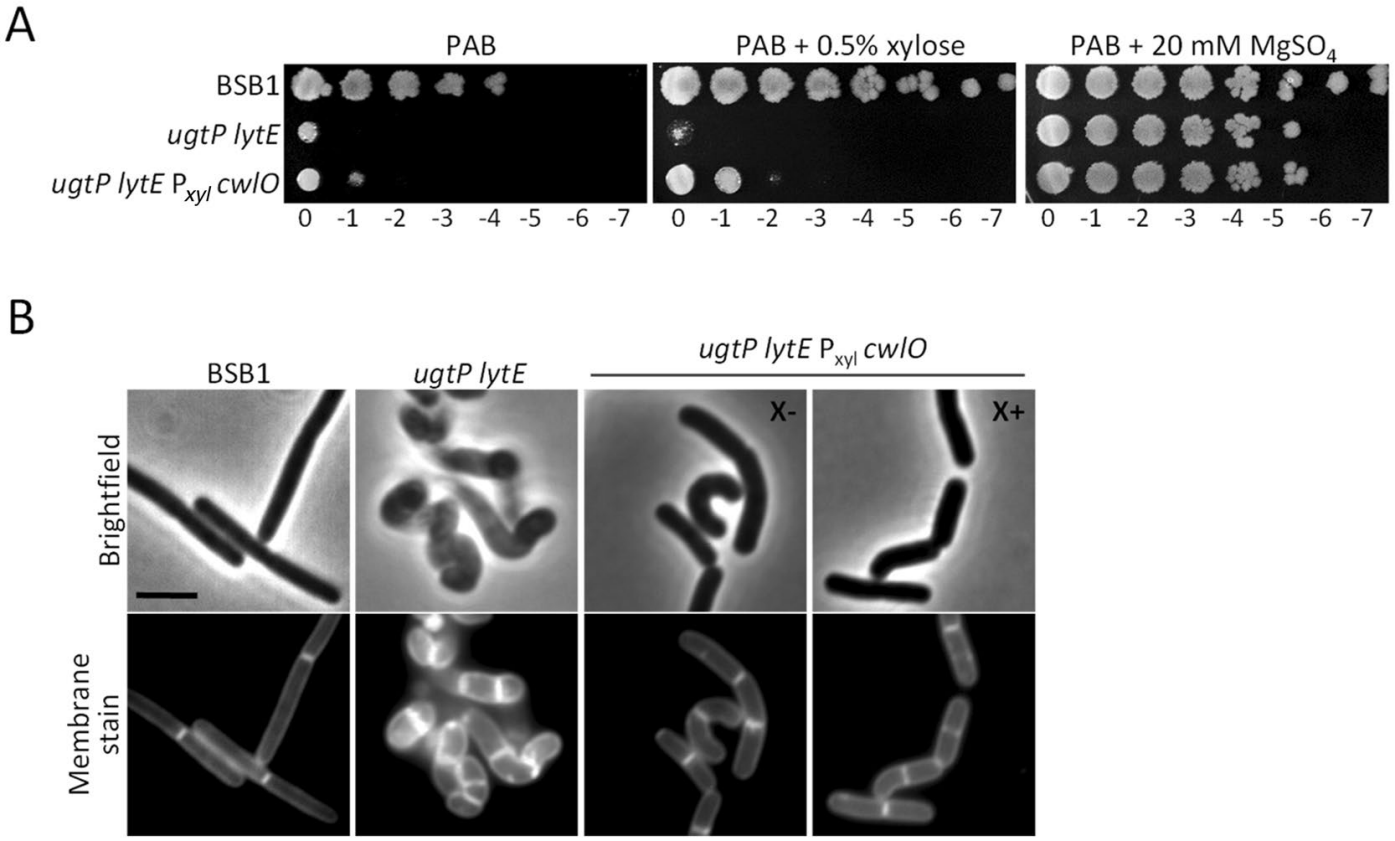
The overexpression of CwlO improved the viability and rod morphology of the *ugtP lytE* mutant. (**A**) Spot plate assay for cells with an additional *cwlO* gene under the control of a xylose promoter. BSB1 Δ*ugtP* Δ*lytE* cells overexpressing CwlO (+xylose) were viable on PAB. (**B**) Fluorescence microscopy for BSB1 Δ*ugtP* Δ*lytE* cells overexpressing CwlO recovered partially the rod-shape morphology however cells were still bent. *X*− without xylose, *X+* with xylose. Scale bar 3 µm.

The depletion of CwlO and LytE was accompanied by round and bent cells and eventually cell death^16,17^ similar to what we observed for BSB1 Δ*ugtP* Δ*lytE* (Fig. 2). Hence, we hypothesised that the loss of rod-shape in our mutants was partially caused by the low peripheral endopeptidase activity. To test this hypothesis, we introduced an extra copy of the *cwlO* gene under the control of a xylose inducible promoter at the *amyE* locus and assessed the phenotype and morphology of the mutant in the presence or absence of xylose. In the presence of xylose, BSB1 Δ*ugtP* Δ*lytE* P_*xyl*_* cwlO* grew moderately better on PAB agar plates, and the addition of magnesium sulphate rescued the conditional lethality of BSB1 Δ*ugtP* Δ*lytE* and the compromised growth of BSB1 Δ*ugtP* Δ*lytE* P_*xyl*_* cwlO* mutant (Fig. 3A). Interestingly, BSB1 Δ*ugtP* Δ*lytE* P_*xyl*_* cwlO* partially recovered the wt cell morphology in the presence or absence of xylose suggesting that the leaky expression of *cwlO* was sufficient to improve cell shape (Fig. 3B, Supplementary Table S4). Cells partially recovered rod-shape but were still occasionally bent. The reason for the partial amelioration of the cell morphology could be that the cofactors of CwlO (FtsEX, SweCD) were not overexpressed^18–20^. Muropeptide analysis from Δ*ugtP* Δ*lytE* cells showed higher levels of endopeptidase activity compared to BSB1 suggesting that the observed defects are not caused by an overall low activity. However, since LytE and CwlO are the only known dl-endopeptidases that localise to the cell periphery, our mutant analysis suggest that the morphological defect caused by deletion of *ugtP* and *lytE* could be due to low activity at the cell periphery. Alternatively, the higher levels of endopeptidase products could be the result of an accumulation of “old”, partially degraded PG material on the outside of the cell, consistent with the rough cell surface observed for these mutants by TEM.

### LytE is dispensable in cells lacking LTA synthases or the glucose transferases TagE and GgaAB

It has been suggested that UgtP has two physiological roles in the cell, as metabolic sensor governing cell size and as glucosyltransferase participating in the synthesis of the LTA^31,40^. During LTA synthesis, UgtP catalyses the addition of two glucose residues to the diacylglycerol moiety (Fig. 1A). Subsequently, LTA synthases catalyse the addition of glycerol residues to the diglucosyl-diacylglycerol^29,49^. *B. subtilis* has three LTA synthases (LtaS, YfnI and YqgS) and one LTA primase (YvgJ)^29^. Thus, in the absence of UgtP, a change in LTA structure or abundance could result in an inefficient CwlO enzyme. To address this possibility, we combined deletions of LTA synthase genes with the *lytE* deletion. Cells lacking individual LTA synthases grew like the wild type in the presence or absence of LytE (Supplementary Fig. S4A). Cells lacking both major LTA synthases, LtaS and YfnI had lower fitness, and the additional deletion of *lytE* did not aggravate the growth defect. The LTA synthase triple mutant BSB1 Δ*ltaS* Δ*yfnI* Δ*yqgS* had similar phenotype as the double mutant BSB1 Δ*ltaS* Δ*yfnI*, and the deletion of *lytE* in both background strains had no additional effect on growth. Microscopic examination of all mutants showed that single LTA synthase mutants lacking LytE were rod-shaped similar to wild-type cells (Supplementary Fig. S4B, Supplementary Table S4). BSB1 Δ*ltaS* Δ*yfnI* showed ~ 10% bent cells compared to ~ 40% with the additional loss of LytE, however the majority of cells were able to maintain rod-shape (Supplementary Table S4). The morphology of BSB1 Δ*ltaS* Δ*yfnI* Δ*yqgS* showed longer cells compared to BSB1 Δ*ltaS* Δ*yfnI* and an increased frequency of bent cells (~ 30%). BSB1 Δ*ltaS* Δ*yfnI* Δ*yqgS* Δ*lytE* exhibit a chaining phenotype with the highest levels of bent cells (~ 55%), however, the cell shape defects were not comparable to those in BSB1 Δ*ugtP* Δ*lytE* and the majority of cells were rod-shaped with a normal average cell length of 3.5 μm (Supplementary Fig. S4B, Supplementary Table S4). We conclude that the LTA synthases are less important than UgtP for the viability and cell morphology of the *lytE* mutant, suggesting that an altered amount or structure of LTA is not the main reason behind the poor function of CwlO in the *ugtP* mutant.

Since *B. subtilis* has three known sugar transferases, UgtP, TagE and GgaAB, responsible for adding glucose residues to LTA or WTAs, we investigated whether these glucosylation enzymes were important for a functional CwlO. Mutants lacking *tagE*, *ggaAB* and/or *lytE* had similar fitness and cell shape as wild type cells, suggesting that CwlO is functional in cells lacking WTA glucosylation or the minor WTA (Supplementary Fig. S5). As a result, it remains unclear if alterations in glucolipid levels, inhibitions of FtsZ polymerisation, or both is the main reason behind the malfunctioning of CwlO.

### Only the MreB actin isoform is required for cell elongation in the ***ugtP*** mutant

**Figure 4:**
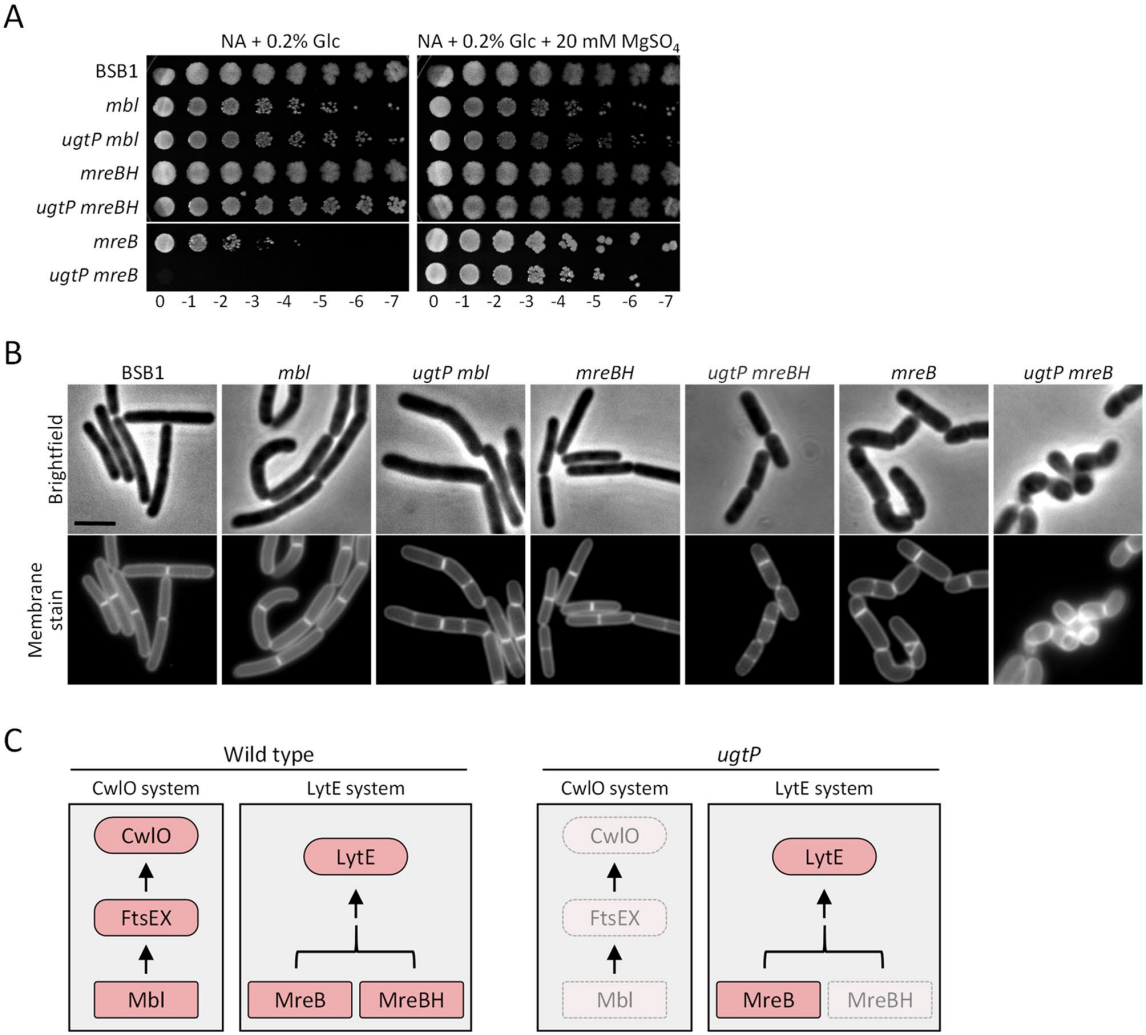
The deletion of *ugtP* in cells lacking MreB homologues. (**A**) Spot plate assay for single or double mutants lacking UgtP and/or MreB homologues. Only the *ugtP mreB* double mutant showed lethality when cells were grown on nutrient agar supplemented with 0.2% glucose. Media supplemented with magnesium recovered the phenotype of the mutants. (**B**) Fluorescence microscopy of mutants grown in LB with 20 mM magnesium sulphate showed a loss of the rod-shape and severe shape defects only for BSB1 Δ*ugtP* Δ*mreB* cells. Scale bar 3 µm. (**C**) The two proposed distinct pathways for the lateral PG hydrolysis during cell elongation in wild type and BSB1 Δ*ugtP* cells^18^.

The MreB homologues, MreB, Mbl or MreBH, control the synthesis and hydrolysis of PG presumably through multiple protein–protein interactions within the elongasome complex^50–52^ (Fig. 4C). MreBH interacts with LytE and ensures its localisation at the lateral cell wall, but the relevance of this interaction or localization requirement remains unclear^19,21^. Double mutant analysis showed that MreB is important for LytE action whereas Mbl is crucial for the FtsEX/CwlO system^18,19^. Since the poor function of CwlO in BSB1 Δ*ugtP* cells is unlikely to be caused by alterations in the LTA synthesis (Supplementary Fig. S4), we tested whether the link between CwlO and the MreB cytoskeleton was disturbed. To this end, we deleted the individual *mreB* isoforms in cells lacking *ugtP* and the corresponding mutant strains were analysed phenotypically and morphologically. Since *mreB* or *mbl* mutants cannot grow on PAB, LB plates supplemented with glucose were used to assess their growth. BSB1 Δ*ugtP* Δ*mbl* grew like the single mutant BSB1 Δ*mbl*, presumably due to the sufficient presence of active LytE in both Δ*ugtP* and Δ*mbl* mutants (Fig. 4A,C). BSB1 Δ*ugtP* Δ*mreBH* and BSB1 Δ*mreBH* grew like wild type despite MreBH having a role in controlling the activity of LytE^19,21^. Conversely, BSB1 Δ*mreB* showed compromised growth and BSB1 Δ*ugtP* Δ*mreB* did not grow at all under these conditions (Fig. 4A). The addition of magnesium sulphate permitted growth of the *ugtP mreB* mutant. Since cells lacking Mbl or MreB were sick we supplemented the growth media with 20 mM magnesium sulphate to investigate possible shape defects. Cells with single deletion of the *mbl*, *mreB* or *mreBH* genes showed mild shape defects compared to wild type cells but all mutants maintained rod-shape (Fig. 4B, Supplementary Table S4). BSB1 Δ*ugtP* Δ*mreBH* and BSB1 Δ*ugtP* Δ*mbl* cells were shorter than wild type, which could be attributed to the loss of *ugtP,* however, most cells maintained rod-shape (Supplementary Table S4). Interestingly, BSB1 Δ*ugtP* Δ*mreB* was synthetically lethal and cells had severe shape defects and loss of rod-shape, similar to BSB1 Δ*cwlO* Δ*lytE* and BSB1 Δ*ugtP* Δ*lytE*. In the light of the partial functional redundancy of the Mre cytoskeletal proteins^53,54^, these results suggest that, in the absence of *ugtP,* the CwlO system is poorly functional and insufficient to support cell elongation and survival (Fig. 4C).

### Reduced PG synthesis allowed growth of the ***ugtP lytE*** mutant

**Figure 5:**
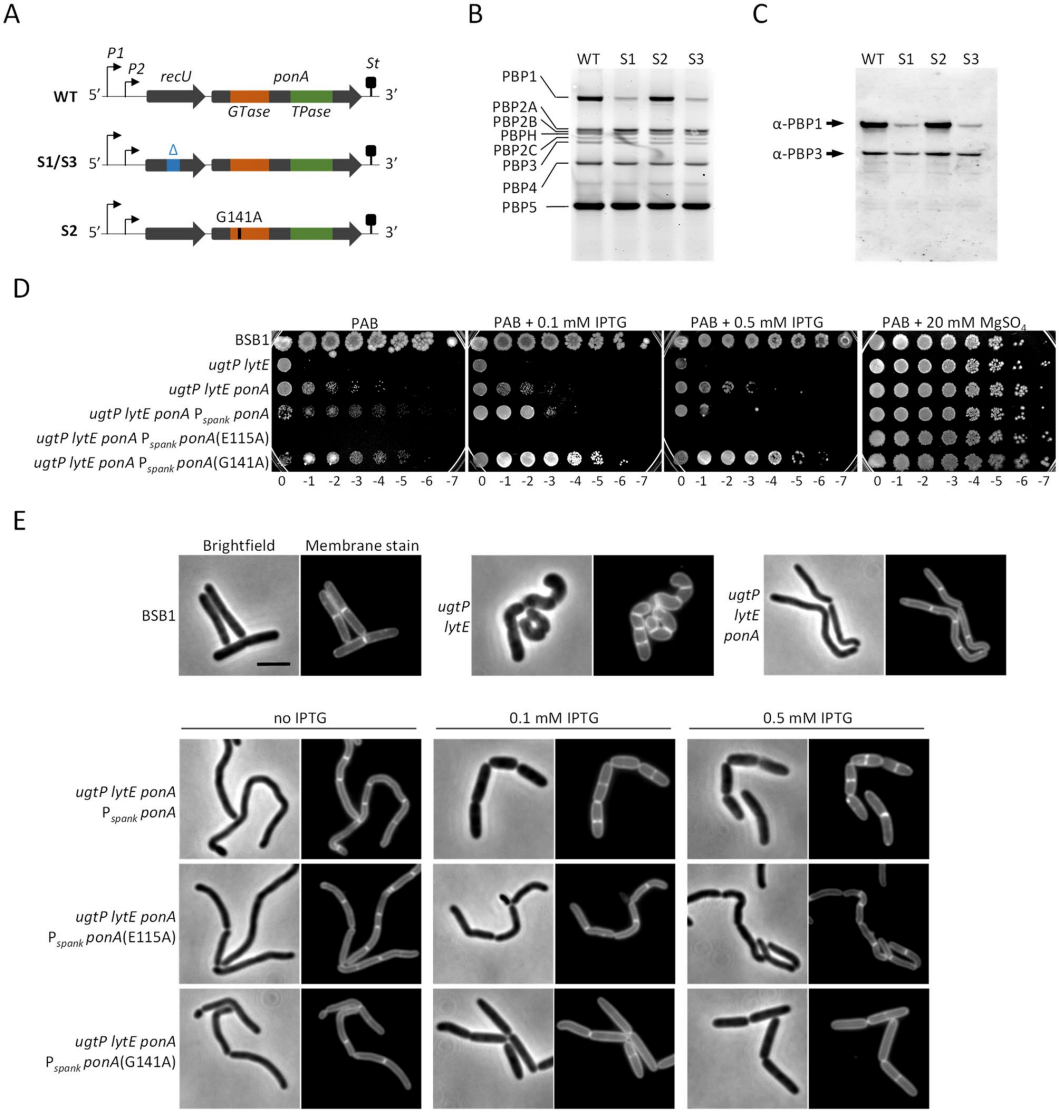
Changes in the PBP1 activity levels allowed growth of the *ugtP lytE* mutant. (**A**) The scheme represents the *ponA* operon. *P1* and *P2* represent the 2 promoters in the operon while *St* represents the stop codon. WT, the codon composition in wild type cells. S1 and S3 represent the two isolated suppressors with alterations in the *recU* sequence. S2 represents the third suppressor with a G141A point mutation in the PBP1 encoding gene *ponA*. (**B**) Bocillin binding assay shows smaller band corresponding to PBP1 for the suppressors S1 and S3 compared to wild type. Bands corresponding to other PBPs were comparable for the three suppressors and wild type cells. (**C**) Western blot against cell lysates from suppressor mutants and wild type cells using PBP1 polyclonal antibody and PBP3 polyclonal antibody as a loading control. The S1 and S3 samples showed smaller bands corresponding to the levels of PBP1 compared to the S2 mutant and WT cells suggesting lower levels of PBP1 in the cells. (**D**) Spot plate assay for mutants with deletions in the *ugtP, lytE* and *ponA* genes, and with ectopic expression of PBP1, PBP1 E115A or PBP1 G141A. *UgtP lytE ponA* triple mutants expressing low levels of PBP1 or PBP1 G141A, showed better phenotype compared to both BSB1 Δ*ugtP* Δ*lytE* and BSB1 Δ*ugtP* Δ*lytE* Δ*ponA*. (**E**) Fluorescence microscopy for the aforementioned mutants showed that BSB1 Δ*ugtP* Δ*lytE* Δ*ponA* recovered the rod-shape, however, cells were thin and bent. BSB1 Δ*ugtP* Δ*lytE* Δ*ponA* expressing low levels of PBP1 or PBP1 G141A, showed amelioration in the cell morphology where mutants looked mostly like the *ugtP* single mutant previously described as short cells (Supplementary Fig. S2). Scale bar 3 µm.

Our data so far suggest that CwlO is functioning poorly in the *ugtP lytE* mutant, resulting in growth and shape defects due to low peripheral endopeptidase activity. Using an unbiased approach to identify mutants that supressed defects caused by low endopeptidase activity, we isolated and studied spontaneous mutants in the *ugtP lytE* background. For this we exploited the fact that the growth of BSB1 Δ*ugtP* Δ*lytE* on PAB agar plates was significantly compromised at 37 °C. Consequently, plates inoculated with this strain were found to exhibit little bacterial growth after 16 h incubation, but after 2–3 days, a number of small colonies, presumably suppressor mutants, were seen to appear. To avoid the emergence of suppressors with accumulated mutations in the genome and to isolate suppressors with improved growth, plates of the *ugtP lytE* mutant were incubated at 45 °C overnight to accelerate growth and increase the selective pressure. Three independently isolated suppressor mutants were chosen and their genomes were sequenced using MiSeq, and the data analysed using CLC genomics software. Two of the suppressor strains (S1 and S3) had insertion deletion mutations in the *recU* gene located immediately upstream of *ponA*, which encodes the class A penicillin binding protein PBP1. The remaining suppressor mutant (S2) had a point mutation in *ponA* that is predicted to replace glycine 141 by alanine in the GTase domain of PBP1 (Fig. 5A). Additionally, all suppressor mutants had a similar insertion deletion mutation in the secreted arabinofuranosidase *abfA* involved in the degradation of arabinan, and the suppressor mutants S2 and S3 had each 2 different missense mutations in the *uvrX* gene encoding a putative UV-damage repair protein. It is unlikely that these mutations contributed to the suppression of the *ugtP lytE* phenotype, therefore they are not further discussed.

There is no crystal structure available for PBP1, but its GTase domain shows 46% identity and 67% homology to that of PBP2 from *S. aureus*, which has a known crystal structure (Supplementary Fig. S6B). Using Phyre^2^ we modelled the structure of PBP1, using *S. aureus* PBP2 as template, and analysed the model structure with PyMOL (Supplementary Fig. S6A)^55^. This analysis showed Gly141 to be located on a conserved loop (residues 139–147) near the GTase active site, hence the G141A mutation might affect the catalytic activity of PBP1, potentially by changing the accessibility of lipid II to the GTase catalytic residue E115. Alternatively, or additionally, the G141A mutation could affect an interaction of PBP1. We next performed a Bocillin-binding assay to test if the expression level of PBP1 or other PBPs were affected in the suppressor mutants. Bocillin is a fluorescently labelled β-lactam that binds to the TPase domain of PBPs. The suppressor mutants S1 and S3, with deletion in the *recU* gene, exhibited a decreased fluorescent signal corresponding to Bocillin-PBP1, but the signals of other Bocillin-PBP bands were unchanged, suggesting that the mutation in *recU* has a polar effect on the expression of PBP1 (Fig. 5B,C). This hypothesis was confirmed by Western blot using ⍺-PBP1 polyclonal antibody and ⍺-PBP3 antibody as a loading control. In contrast, the abundance of PBP1(G141A) in suppressor S2 was comparable to that of PBP1 in wild-type (Fig. 5B,C).

To test for a role of PBP1 level or activity in these suppressor mutations, we deleted the *ponA* gene in BSB1 Δ*ugtP* Δ*lytE*, and expressed PBP1, PBP1(G141A) or PBP1(E115A) (with predicted inactive GTase) ectopically from the *amyE* locus. BSB1 Δ*ugtP* Δ*lytE* Δ*ponA* partially recovered viability and growth on PAB plates, and cells recovered rod-shape morphology, although the cells were thin and bent (Fig. 5D,E, Supplementary Table S4). BSB1 Δ*ugtP* Δ*lytE* Δ*ponA* P_*spank*_* ponA* showed improved growth at low inducer levels (0.1 mM IPTG) but its growth was strongly impaired in the presence of high inducer levels (0.5 mM IPTG). BSB1 Δ*ugtP* Δ*lytE* Δ*ponA* P_*spank*_* ponA*(E115A) was not viable on PAB even in the presence of inducer and cells had similar morphology to BSB1 Δ*ugtP* Δ*lytE* Δ*ponA* when grown in LB. Interestingly, the fitness of BSB1 Δ*ugtP* Δ*lytE* Δ*ponA* P_*spank*_* ponA*(G141A) improved dramatically in the presence of low and high inducer levels and cells showed similar viability to wild type. Moreover, the morphology of the mutant also improved significantly upon the expression of PBP1(G141A) and cells regained their wild type rod-shape (Fig. 5D,E, Supplementary Table S4). Collectively, these results suggest that the growth of the *ugtP lytE* mutant can be significantly improved by reducing the abundance or activity of PBP1.

## Discussion

Previous work showed that the conserved glucolipid biosynthesis pathway functions as a metabolic sensor that couples nutrient availability to cell division^40^. UgtP, a key element in this pathway, is widely conserved in Gram-positive bacteria and was described to coordinate cell growth, by controlling Z-ring formation, with nutrient availability^40^. UgtP also transfers two glucose residues to the diacylglycerol moiety which acts as the membrane linker for LTA. Due to the observed high levels of transcriptional cell wall stress response in the *ugtP* mutant^37^, we initially set out to determine how the loss of UgtP impacted PG synthesis. From this analysis the phenotypes of the *ugtP* mutant combined with mutations in other key cell wall metabolism genes has allowed us to dissect out aspects of the interplay between PG synthesis and hydrolysis.

The depletion of LytE and CwlO results in low endopeptidase activity at the cell periphery and eventually cell death^16,17^. Additionally, the growth of *mbl* mutant cells on PAB was rescued by overexpressing the sigma factor *sigI*, or by deleting *ltaS*^49,56^. Since *lytE* has a *sigI*-dependent promoter, both suppressors rescued the *mbl* mutant presumably by overexpressing LytE to provide sufficient endopeptidase activity at the cell periphery to allow cells to propagate^25,49,56^. Alternatively, the overexpression of MreBH, which is also *sigI*-dependent, could have a role in rescuing the growth of *mbl* mutant cells. Interestingly, the *ugtP lytE* mutant cells were sick and unable to grow on PAB plates, in sharp contrast to *ugtP cwlO* mutant cells, suggesting that in the absence of *ugtP* cells depend mostly on LytE for elongation (Fig. 2A, Supplementary Table S4). Despite MreBH's role in localizing LytE in wt cells^21^, our data suggest that LytE function relies mostly on MreB in the *ugtP* mutant. This interpretation is based on the observation that the additional deletion of *ugtP* in the *mbl* or *mreBH* mutant cells did not aggravate the cell phenotype and the majority of cells maintained rod-shape but the *ugtP mreB* mutant had severe growth defect and loss of rod-shape, phenocopying the *cwlO lytE* or *ugtP lytE* double mutants (Fig. 4C).

**Figure 6:**
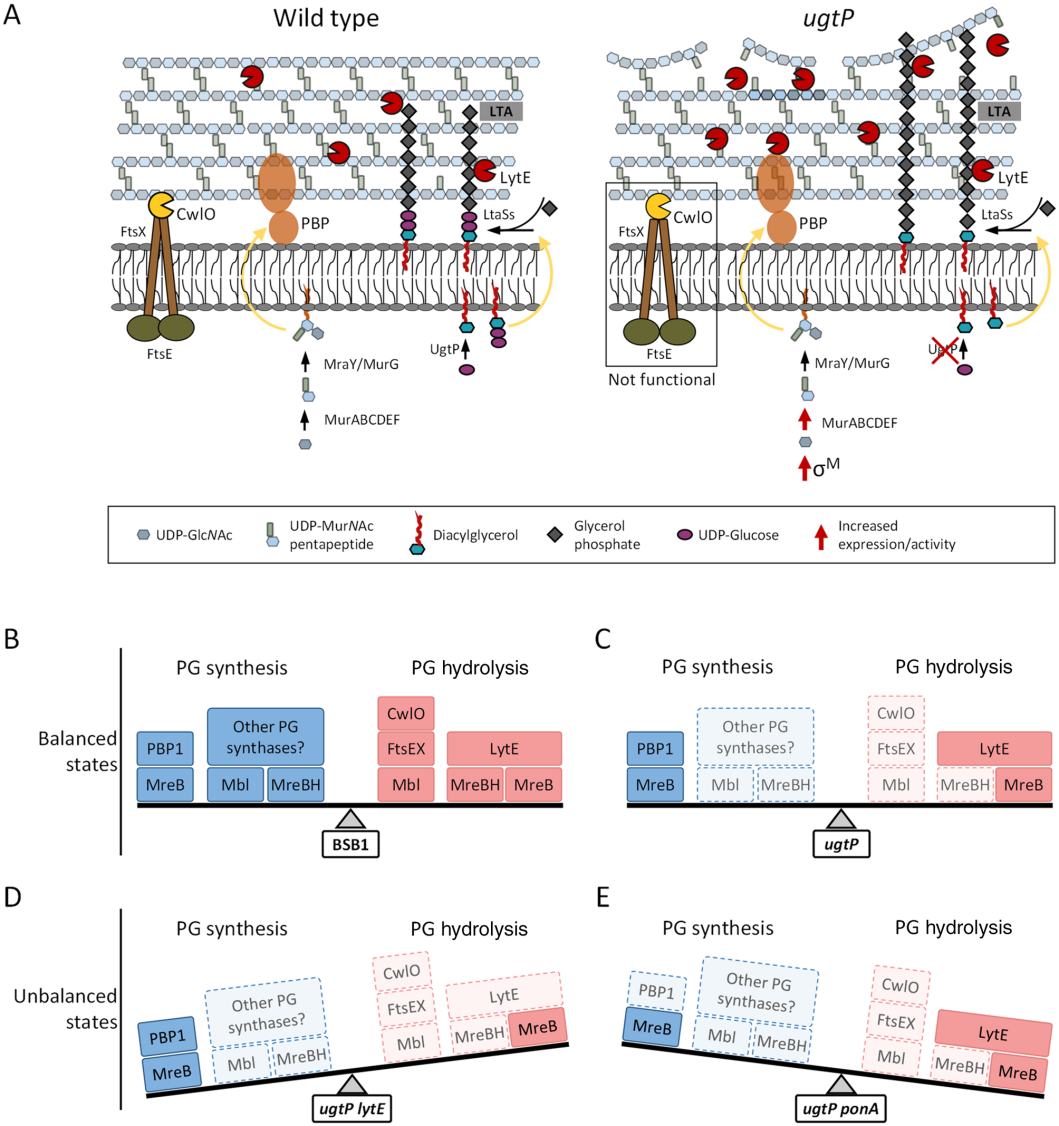
Schematic representation of the cell wall changes occurring in the *ugtP* mutant. (**A**) The genetic evidence in this work suggests that in comparison to wild type cells (left panel), cells lacking UgtP (right panel) showed lower CwlO activity, an increase in the PG precursor levels resulting in an upturn in PG synthesis and an increase in the LytE endopeptidase activity. These results support the idea that the ugtP mutant maintain its cell wall integrity and rod-shape by balancing the increased PG synthesis (PBP1) and hydrolysis (LytE). A representation of the proteins involved in peripheral PG synthesis or hydrolysis in wild type cells and the mutants studied in this work are shown in panels (**B**–**E**). Dark coloured boxes represent functioning proteins and light coloured boxes represent malfunctioning proteins. (**B**) BSB1 cells had functional proteins resulting in balanced PG synthesis and hydrolysis. (**C**) Δ*ugtP* cells exhibited a malfunction in several proteins involved in PG synthesis and/or hydrolysis, such that the cell maintains a healthy balance between PG synthesis and hydrolysis. (**D**) Δ*ugtP* Δ*lytE* cells and (**E**) Δ*ugtP* Δ*ponA* cells showed a malfunction in several proteins however, these mutants exhibited a misbalance in PG synthesis and/or hydrolysis resulting in loss of rod-shape and cell death. Therefore, a balanced synthesis and hydrolysis, potentially by the same cytoskeleton system, is important for PG synthesis and cell shape.

The *ugtP* mutant exhibited rough cell surface and altered PG structure suggesting higher or uncontrolled cell wall hydrolase activity along the cell periphery. This observation is consistent with previously published data showing increased levels of the endopeptidase LytE in cells grown in the absence of UgtP (Fig. 6A)^25^. The increased autolysis activity was paralleled by the induction of the extracellular sigma factor SigM, which responds to cell wall synthesis and cell shape maintenance, and increased levels of some PG precursors (Fig. 6A, Supplementary Fig. S1)^44,57,58^. To explain the observations reported in this paper, we propose a model according to which PG synthases and hydrolases work together to ensure a safe enlargement of the PG (Fig. 6B). In wild type BSB1 cells, all proteins are functional thus creating a balance between PG synthesis and hydrolysis and maintaining rod-shape. In the *ugtP* mutant cells, the functions of several cell wall proteins, including PG synthases and hydrolases, are impaired such that the cell maintains rod-shape and a healthy balance between PG synthesis and hydrolysis, albeit the new balance is less robust (Fig. 6C). The removal of *lytE* from the *ugtP* mutant cells resulted in sick cells and a loss of rod-shape, by generating a misbalance towards enhanced synthetic activity, caused by reduced activity of the CwlO autolytic system (Fig. 6D). By contrast, it was not possible to delete *ponA* in the background of the *ugtP* mutant even when the growth medium was supplemented with magnesium sulphate, presumably due to misbalanced system towards enhanced autolytic activity (Fig. 6E). Interestingly, the deletion of *ponA* was possible in the *ugtP lytE* mutant cells, and the *ugtP lytE ponA* triple mutant cells partially recovered growth on PAB and rod morphology. These results show that several proteins that are dispensable in wild-type cells, such as LytE and PBP1, become essential in the absence of UgtP when synthase or hydrolase activities become unbalanced. Consequently, maintaining a carefully balanced PG synthesis and hydrolysis is essential for cells lacking UgtP. Nonetheless, the reason for such changes in synthesis and hydrolysis in the *ugtP* mutant remain unclear. The loss of UgtP function resulted in premature septal formation and shorter cells. Such changes in cell morphology could require a readjustment in the PG synthesis/hydrolysis machinery to maintain cell integrity, which could explain the alterations observed in the activity/function of multiple enzymes in this study.

Altogether, our work starts to unravel the intricate balance between PG synthesis and hydrolysis which must be sufficiently robust to allow cells to propagate when some of the components fail to function (in a deletion mutant or certain environments). Most likely, this robustness is achieved by the seeming redundancies in PG enzymes and mechanisms by which they are coordinated.

## Materials and methods

### General methods

*Bacterial strains, plasmids, and growth conditions.* Bacterial strains and plasmids used in this work are listed in Supplementary Table S1 in the Supplementary Information. Primers are listed in Supplementary Table S2. *B. subtilis* or *E. coli* cells were cultivated in Luria Britani (LB), Difco antibiotic medium no. 3 (PAB) or competence medium depending on the requirements of the experiments. Fresh cultures were inoculated with overnight culture and grown at 30 or 37 °C with continuous shaking. For solid media, we used either nutrient agar (Oxoid) or added 1% agar (Bacteriological agar no. 1, Oxoid) to the indicated liquid media containing the appropriate supplements (chloramphenicol 5 μg/ml, erythromycin 1 μg/ml, ampicillin 5 μg/ml, kanamycin for *B. subtilis* 2–5 μg/ml and for *E. coli* 50 μg/ml, tetracycline 10 μg/ml, IPTG 0.1–1 mM and MgSO_4_ 20 mM).

#### Spot plate assay

Cell cultures with OD_600_ 1.0 were serially diluted in 96 well plates using fresh LB media followed by agar plate inoculation using a multipoint inoculator. Assays were replicated at least twice.

### Bocillin assay

Bocillin Assay was adopted from a published protocol^59^ with minor modifications as in Sassine et al.^60^. Samples were analysed by SDS-PAGE and the final gel was scanned with a Typhoon scanner.

### Western blot

Western blotting was done as described previously^61^, using rabbit polyclonal antibodies raised against purified PBP1 and PBP3 proteins as primary antibodies and goat anti-rabbit as secondary antibodies.

### Cloning of strains and plasmids

Genomic DNA was purified using DNA purification kit (Sigma) as per manufacturer's instructions. *B. subtilis* cells were transformed, with purified genomic DNA or PCR products, following the method described by Anagnostopoulos and Spizizen with modifications according to Hamoen et al.^62,63^. Agar plates with corresponding antibiotics and Mg ions were used for selection.

Plasmids were cloned using restriction endonuclease RE enzymes. For the construction of pJS01 plasmid, first *ugtP* was amplified by PCR using the JS05 and JS06 nucleotides. DNA fragments and pDR111 plasmid were digested using SalI and SpHI RE and ligated using T4 DNA ligase. For the construction of pJS03 plasmid, *gtaB* was amplified by PCR using the JS09 and JS010 nucleotides. DNA fragments and pAPNC213 plasmid were digested using SalI and EcoRI RE and ligated using T4 DNA ligase.

### Microscopy

Microscopic images were taken using inverted Nikon Eclipse Ti microscope coupled to a Sony Cool-Snap HQ2 CCD camera (Roper scientific) operated by Metamorph 6 imaging software (Universal imaging). Cells were grown in LB media and images were taken during exponential phase (OD_600_ 0.5). Samples (0.5 μl) were mounted on 1% agarose and imaging was performed with brightfield illumination with 300 ms exposure time. For membrane staining, cells were mounted on agarose with Nile red dye (1 μg/ml, Molecular Probes). All images were analysed with ImageJ^64^.

### Transmission electron microscopy

Samples were dehydrated and processed by Kathryn White at the electron microscopy unit at Newcastle University. Cells were grown in LB media to OD_600_ 0.5 at 37 °C, mixed in a 1:1 ratio with fix buffer I (2% glutaraldehyde, 0.1 M sodium cacodylate) and incubated at 4 °C for overnight. Cells were pelleted then washed with 0.1 M sodium cacodylate buffer followed by a secondary fixation step with 1% osmium tetroxide in water for 1 h. Cells were dehydrated using graded acetone (25%, 50%, 75% and 100%). Subsequently, cells were impregnated sequentially in 25%, 50%, 75% and 100% resin. A final embed was performed in 100% resin at 60 °C for 24–36 h. Ultrathin sections of 70 nm were then cut with a diamond knife using a Leica EM UC7 ultramicrotome. Sections were stretched with chloroform to eliminate compression, transferred to a Pioloform-filmed copper grids and stained on a Leica EM AC20 automatic staining machine with 2% aqueous uranyl acetate 3% lead citrate. A Philips CM 100 Compustage (FEI) Transmission Electron Microscope coupled to an AMT CCD camera (Deben) was used to collect images for several mutant cells.

The methods for cell wall purification and muropeptide analysis, and metabolomics can be found in the supplemental material.

## Supplementary information

Supplementary Information

## Abbreviations

PBP: Penicillin-binding protein
PG: Peptidoglycan
TPase: Transpeptidase
GTase: Glycosyltransferase

## References

[1] Vollmer W, Blanot D, de Pedro MA (2008). Peptidoglycan structure and architecture. FEMS Microbiol. Rev..

[2] Bhavsar AP, Brown ED (2006). Cell wall assembly in *Bacillus subtilis*: How spirals and spaces challenge paradigms. Mol. Microbiol..

[3] Lovering AL, Safadi SS, Strynadka NCJ (2012). Structural perspective of peptidoglycan biosynthesis and assembly. Annu. Rev. Biochem..

[4] Barrett D (2007). Analysis of glycan polymers produced by peptidoglycan glycosyltransferases. J. Biol. chermistry.

[5] Meeske AJ (2016). SEDS proteins are a widespread family of bacterial cell wall polymerases. Nature.

[6] Pedersen LB, Angert ER, Setlow P (1999). Septal localization of penicillin-binding protein 1 in *Bacillus subtilis*. J. Bacteriol..

[7] Scheffers D-J, Errington J (2004). PBP1 is a component of the *Bacillus subtilis* cell division machinery. J. Bacteriol..

[8] Claessen D (2008). Control of the cell elongation-division cycle by shuttling of PBP1 protein in *Bacillus subtilis*. Mol. Microbiol..

[9] Kawai Y, Daniel RA, Errington J (2009). Regulation of cell wall morphogenesis in *Bacillus subtilis* by recruitment of PBP1 to the MreB helix. Mol. Microbiol..

[10] Murray T, Popham DL, Setlow P (1998). *Bacillus subtilis* cells lacking penicillin-binding protein 1 require increased levels of divalent cations for growth. J. Bacteriol..

[11] Pazos M, Peters K, Vollmer W (2017). Robust peptidoglycan growth by dynamic and variable multi-protein complexes. Curr. Opin. Microbiol..

[12] Singh SK, Saisree L, Amrutha RN, Reddy M (2012). Three redundant murein endopeptidases catalyse an essential cleavage step in peptidoglycan synthesis of *Escherichia coli* K12. Mol. Microbiol..

[13] Smith TJ, Blackman SA, Foster SJ (1996). Peptidoglycan hydrolases of *Bacillus subtilis* 168. Microb. Drug Resist..

[14] Smith TJ, Blackman SA, Foster SJ (2000). Autolysins of *Bacillus subtilis*: Multiple enzymes with multiple functions. Microbiology.

[15] Banzhaf M (2012). Cooperativity of peptidoglycan synthases active in bacterial cell elongation. Mol. Microbiol..

[16] Bisicchia P (2007). The essential YycFG two-component system controls cell wall metabolism in *Bacillus subtilis*. Mol. Microbiol..

[17] Hashimoto M, Ooiwa S, Sekiguchi J (2012). Synthetic lethality of the *lytE cwlO* genotype in *Bacillus subtilis* is caused by lack of d,l-endopeptidase activity at the lateral cell wall. J. Bacteriol..

[18] Dominguez-Cuevas P (2013). Differentiated roles for MreB-actin isologues and autolytic enzymes in *Bacillus subtilis* morphogenesis. Mol. Microbiol..

[19] Meisner J (2013). FtsEX is required for CwlO peptidoglycan hydrolase activity during cell wall elongation in *Bacillus subtilis*. Mol. Microbiol..

[20] Brunet YR, Wang X, Rudner DZ (2019). SweC and SweD are essential co-factors of the FtsEX-CwlO cell wall hydrolase complex in *Bacillus subtilis*. PLoS Genet..

[21] Carballido-Lopez R (2006). Actin homolog MreBH governs cell morphogenesis by localization of the cell wall hydrolase LytE. Dev. Cell.

[22] Yamamoto H, Kurosawa SI, Sekiguchi J (2003). Localization of the vegetative cell wall hydrolases LytC, LytE, and LytF on the *Bacillus subtilis* cell surface and stability of these enzymes to cell wall-bound or extracellular proteases. J. Bacteriol..

[23] Ohnishi R, Ishikawa S, Sekiguchi J (1999). Peptidoglycan hydrolase *lytF* plays a role in cell separation with cwlf during vegetative growth of *Bacillus subtilis*. J. Bacteriol..

[24] Percy MG, Gründling A (2014). Lipoteichoic acid synthesis and function in gram-positive bacteria. Annu. Rev. Microbiol..

[25] Kasahara J (2016). Teichoic acid polymers affect expression and localization of dl-endopeptidase LytE required for lateral cell wall hydrolysis in *Bacillus subtilis*. J. Bacteriol..

[26] Perego M (1995). Incorporation of D-alanine into lipoteichoic acid and wall teichoic-acid in *Bacillus subtilis*—identification of genes and regulation. J. Biol. Chem..

[27] Wecke J, Perego M, Fischer W (1996). d-Alanine deprivation of *Bacillus subtilis* teichoic acids is without effect on cell growth and morphology but affects the autolytic activity. Microb. Drug Resist. Epidemiol. Dis..

[28] Wecke J, Madela K, Fischer W (1997). The absence of D-alanine from lipoteichoic acid and wall teichoic acid alters surface charge, enhances autolysis and increases susceptibility to methicillin in *Bacillus subtilis*. Microbiology.

[29] Wormann ME, Corrigan RM, Simpson PJ, Matthews SJ, Gründling A (2011). Enzymatic activities and functional interdependencies of *Bacillus subtilis* lipoteichoic acid synthesis enzymes. Mol. Microbiol..

[30] Jorasch P, Wolter FP, Zahringer U, Heinz E (1998). A UDP glucosyltransferase from *Bacillus subtilis* successively transfers up to four glucose residues to 1,2-diacylglycerol: Expression of *ypfP* in *Escherichia coli* and structural analysis of its reaction products. Mol. Microbiol..

[31] Gründling A, Schneewind O (2007). Genes required for glycolipid synthesis and lipoteichoic acid anchoring in *Staphylococcus aureus*. J. Bacteriol..

[32] Schirner K, Errington J (2009). Influence of heterologous MreB proteins on cell morphology of *Bacillus subtilis*. Microbiology.

[33] Yasbin RE, Maino VC, Young FE (1976). Bacteriophage resistance in *Bacillus subtilis* 168, W23, and interstrain transformants. J. Bacteriol..

[34] Matsuoka S (2011). The *Bacillus subtilis* essential gene *dgkB* is dispensable in mutants with defective lipoteichoic acid synthesis. Genes Genet. Syst..

[35] Kawai F, Hara H, Takamatsu H, Watabe K, Matsumoto K (2006). Cardiolipin enrichment in spore membranes and its involvement in germination of *Bacillus subtilis* Marburg. Genes Genet. Syst..

[36] Matsuoka S (2017). Biological functions of glucolipids in *Bacillus subtilis*. Genes Genet. Syst..

[37] Salzberg LI, Helmann JD (2008). Phenotypic and transcriptomic characterization of *Bacillus subtilis* mutants with grossly altered membrane composition. J. Bacteriol..

[38] Seki T, Furumi T, Hashimoto M, Hara H, Matsuoka S (2019). Activation of extracytoplasmic function sigma factors upon removal of glucolipids and reduction of phosphatidylglycerol content in *Bacillus subtilis* cells lacking lipoteichoic acid. Genes Genet. Syst..

[39] Chien AC, Zareh SKG, Wang YM, Levin PA (2012). Changes in the oligomerization potential of the division inhibitor UgtP co-ordinate *Bacillus subtilis* cell size with nutrient availability. Mol. Microbiol..

[40] Weart RB (2007). A Metabolic sensor governing cell size in bacteria. Cell.

[41] Sauer U, Eikmanns BJ (2005). The PEP-pyruvate-oxaloacetate node as the switch point for carbon flux distribution in bacteria. FEMS Microbiol. Rev..

[42] Cronan JE, Waldrop GL (2002). Multi-subunit acetyl-CoA carboxylases. Prog. Lipid Res..

[43] Schujman GE (2006). Structural basis of lipid biosynthesis regulation in Gram-positive bacteria. EMBO J..

[44] Matsuoka S (2011). Abnormal morphology of *Bacillus subtilis ugtP* mutant cells lacking glucolipids. Genes Genet. Syst..

[45] Atrih A, Bacher G, Williamson MP, Foster SJ (1999). Analysis of peptidoglycan structure from vegetative cells of *Bacillus subtilis* 168 and role of PBP 5 in Peptidoglycan maturation. J. Bacteriol..

[46] Bisicchia P, Bui NK, Aldridge C, Vollmer W, Devine KM (2011). Acquisition of VanB-type vancomycin resistance by *Bacillus subtilis*: The impact on gene expression, cell wall composition and morphology. Mol. Microbiol..

[47] Fukushima T (2006). A new d,l-endopeptidase gene product, YojL (renamed CwlS), plays a role in cell separation with LytE and LytF in* Bacillus subtili*s. J. Bacteriol..

[48] Dajkovic A (2017). Hydrolysis of peptidoglycan is modulated by amidation of meso-diaminopimelic acid and Mg^2+^ in *Bacillus subtilis*. Mol. Microbiol..

[49] Schirner K, Marles-Wright J, Lewis RJ, Errington J (2009). Distinct and essential morphogenic functions for wall- and lipo-teichoic acids in *Bacillus subtilis*. EMBO J..

[50] Garner EC (2011). Coupled, circumferential motions of the cell wall synthesis machinery and MreB filaments in *B. subtilis*. Science (80).

[51] Carballido-López R, Errington J (2003). A dynamic bacterial cytoskeleton. Trends Cell Biol..

[52] Dominguez-Escobar J (2011). Processive movement of MreB-associated cell wall biosynthetic complexes in bacteria. Science (80).

[53] Jones LJF (2001). Control of cell shape in bacteria: Helical, actin-like filaments in *Bacillus subtilis*. Cell.

[54] Kawai Y, Asai K, Errington J (2009). Partial functional redundancy of MreB isoforms, MreB, Mbl and MreBH, in cell morphogenesis of *Bacillus subtilis*. Mol. Microbiol..

[55] Lovering AL, De Castro LH, Lim D, Strynadka NCJ (2007). Structural insight into the transglycosylation step of bacterial cell-wall biosynthesis. Science (80).

[56] Schirner K, Errington J (2009). The cell wall regulator σI specifically suppresses the lethal phenotype of mbl mutants in *Bacillus subtilis*. J. Bacteriol..

[57] Cao M, Wang T, Ye R, Helmann JD (2002). Antibiotics that inhibit cell wall biosynthesis induce expression of the *Bacillus subtilis* sigma(W) and sigma(M) regulons. Mol. Microbiol..

[58] Jervis AJ, Thackray PD, Houston CW, Horsburgh MJ, Moir A (2007). SigM-responsive genes of *Bacillus subtilis* and their promoters. J. Bacteriol..

[59] Zhao G, Meier TI, Kahl SD, Gee KR, Blaszczak LC (1999). Bocillin FL, a sensitive and commercially available reagent for detection of penicillin-binding proteins. Antimicrob. Agents Chemother..

[60] Sassine J (2017). Functional redundancy of division specific penicillin-binding proteins in *Bacillus subtilis*. Mol. Microbiol..

[61] Daniel RA, Harry EJ, Errington J (2000). Role of penicillin-binding protein PBP 2B in assembly and functioning of the division machinery of *Bacillus subtilis*. Mol. Microbiol..

[62] Anagnostopoulos C, Spizizen J (1961). Requirements for transformation in *Bacillus Subtilis*. J. Bacteriol..

[63] Hamoen LW, Smits WK, de Jong A, Holsappel S, Kuipers OP (2002). Improving the predictive value of the competence transcription factor (ComK) binding site in *Bacillus subtilis* using a genomic approach. Nucleic Acids Res..

[64] Schneider CA, Rasband WS, Eliceiri KW (2012). NIH Image to ImageJ: 25 years of image analysis. Nat. Methods.

