# Publisher Correction: Cell morphology maintenance in *Bacillus subtilis* through balanced peptidoglycan synthesis and hydrolysis

**DOI:** 10.1038/s41598-021-85465-2

**Published:** 2021-03-11

**Authors:** Jad Sassine, Joana Sousa, Michael Lalk, Richard A. Daniel, Waldemar Vollmer

**Affiliations:** 1 Centre for Bacterial Cell Biology, Biosciences Institute, Faculty of Medical Sciences, Newcastle University, Newcastle upon Tyne, UK; 2 Institute of Biochemistry, University of Greifswald, 17489 Greifswald, Germany; 3 Present Address: Department of Biochemistry, University of Oxford, Oxford, UK; 4 Present Address: Innovayt A/S, Avenida João Paulo II nº 30, 4715‑034 Braga, Portugal

Correction to: *Scientific Reports* 10.1038/s41598-020-74609-5, published online 21 October 2020

In the original version of this Article, Jad Sassine was incorrectly affiliated with ‘Present address: Innovayt A/S, Avenida João Paulo II nº 30, 4715-034 Braga, Portugal’. The correct affiliation is listed below.

Present address: Department of Biochemistry, University of Oxford, Oxford, UK

Joana Sousa was incorrectly affiliated with ‘Present address: Department of Biochemistry, University of Oxford, Oxford, UK’. The correct affiliation is listed below.

Present address: Innovayt A/S, Avenida João Paulo II nº 30, 4715-034 Braga, Portugal

This error has now been corrected in the PDF and HTML versions of the Article.

