# Supplementary material for: Cell morphology maintenance in Bacillus subtilis through balanced peptidoglycan synthesis and hydrolysis
Source: Sci Rep. 2020 Oct 21;10:17910. doi: 10.1038/s41598-020-74609-5 (PMC7578834; doi:10.1038/s41598-020-74609-5)
Supplement: Supplementary file 1 — Supplementary Information [file 41598_2020_74609_MOESM1_ESM.pdf]

SUPPORTING INFORMATION

Cell morphology maintenance in *Bacillus subtilis* through balanced peptidoglycan synthesis and hydrolysis

Jad Sassine[1ǂ], Joana Sousa[2‡], Michael Lalk[2], Richard A. Daniel[1*] and Waldemar Vollmer[1*]

[1]Centre for Bacterial Cell Biology, Biosciences Institute, Faculty of Medical Sciences, Newcastle University, Newcastle upon Tyne, UK.

[2]Institute of Biochemistry, University of Greifswald, Greifswald 17489, Germany.

Keywords: cell wall, peptidoglycan, metabolomics, penicillin-binding proteins, endopeptidase

Abbreviations: PBP, penicillin-binding protein; PG, peptidoglycan; TPase, transpeptidase; GTase: Glycosyltransferase

‡Present address: Innovayt A/S, Avenida João Paulo II nº 30, 4715-034 Braga, Portugal.

ǂ Present address: Department of Biochemistry, University of Oxford, Oxford, UK.

*To whom correspondence may be addressed. E-mail: richard.daniel@ncl.ac.uk and w.vollmer@ncl.ac.uk

INDEX

Supplemental Methods

Supplemental Figures

- Fig. S1: Relative intracellular concentrations of peptidoglycan precursors in BSB1, *pgcA* and *ugtP* mutants.
- Fig. S2: The absence of UgtP, GtaB or PgcA resulted in short cells.
- Fig. S3: The *gtaB* mutant requires LytE to maintain rod-shape.
- Fig. S4: LytE is dispensable for cells lacking LTA synthases.
- Fig. S5: LytE is dispensable for cells lacking the glucose transferases TagE and GgaAB.
- Fig. S6: Ribbon representation of the GTase domain structure of PBP1 modelled to PBP2 from *S. aureus* (SaPBP2B) using Phyre[2].

Supplemental Tables

- Table S1: List of strains and plasmids
- Table S2: List of nucleotides
- Table S3: Relative levels of intracellular metabolites
- Table S4: Cell measurements and morphology
- Table S5: Muropeptide identities and quantification.

References

SUPPLEMENTAL METHODS

Cell wall purification and muropeptide analysis

This method was adopted from Atrih *et al.*, (1999) and modified as per Bisicchia *et al.*, (2011)[1,2]. Culture of *B. subtilis* cells was grown to OD_{600} 0.5 then cooled down to 4˚C. Cells were pelleted (10,000 *g*/ 4˚C/ 15 min) then resuspended in ice-cold 50 mM Tris/HCl, pH 7. Cell suspension was dropped into slightly boiling 5% SDS, then the lysate was cooled down at room temperature overnight. Lysates were centrifuged (12000 *g*/ 30 min/ room temperature), the supernatant was discarded, and the pellet was resuspended in 1 M NaCl. The lysate was washed with 1 M NaCl then H_2O MilliQ until the suspension was free of SDS. The lysate was mixed with glass beads and cells were broken down using a bead beater (Thermo FastPrep FP120). Broken cells were filtered then washed with 10 ml H_2O MilliQ. The filtrate was then centrifuged (2000 *g*/ 5 min/ room temperature). The supernatant was centrifuged again for 30 min at 25000 *g*. The pellet was resuspended in Tris buffer (100 mM Tris/HCl, 20 mM $MgSO_4$, pH 7.5) to which DNase I (10 µg/ml [Sigma]) and RNase (50 µg/ml [Sigma]) were added. The sample was stirred at 37˚C for 2 h followed by the addition of $CaCl_2$ (10 mM), trypsin (Novagen/Merck), porcine pancreas (100 µg/ml) and stirred for another 18 h at 37˚C. SDS (1%) was added and the sample and incubated for 15 min at 80˚C. The mix was then centrifuged (25000 *g*/ 30 min/ room temperature) and the pellet was resuspended in LiCl (8 M). The sample was incubated for 15 min at 37˚C then Centrifuged as above. The pellet was resuspended in EDTA (100 mM and pH 7.0), incubated for 15 min at 37˚C then centrifuged as above. The pellet was washed, resuspended and centrifuged twice with 30 ml H_2O MilliQ, then centrifuged and resuspended in H_2O MilliQ. The suspension was frozen at -80˚C for at least 1 h then lyophilized for 2 days using an Alpha 1-2 freeze dryer (Biopharma).

Cell wall (5 mg) was dissolved in 3 ml hydrofluoric acid at 4˚C for 48 h with stirring. Next, the sample was centrifuged (90000 *rpm*/ 30 min/ 4˚C). The supernatant was discarded and the pellet was washed twice with H_2O MilliQ, once with 100 mM Tris/HCl pH 7.0 and twice with ice-cold H_2O MilliQ, respectively. The murein was resuspended in H_2O MilliQ and stored with sodium azide (0.05%) at 4˚C.

Muropeptides were generated from the digestion of peptidoglycan with cellosyl (Hoechst, Germany) following an established protocol[3]. PG was digested with 8 µg of cellosyl in cellosyl buffer (20 mM NaH_2PO_4, pH 4.8) at 37°C with shaking. Samples were incubated at 100˚C for 7 min and centrifuged at 14,000 *rpm* for 10 min. An equal volume of sodium borate (0.5 M, pH 9.0) was added to samples in addition to a full small spatula of solid sodium borohydride and centrifuged at 4000 *rpm* for 30 min. The pH was adjusted between 3 and 4 with 20% phosphoric acid.

The HPLC analysis was performed using Agilent Technologies Series 1200 HPLC system with a reverse phase column (Prontosil 120-3-C18-AQ 3 µM, Bischoff). A linear gradient was used from 100% solvent A (40 mM sodium phosphate pH 4.5 + 0.0003% sodium azide) to 100% solvent B (40 mM sodium phosphate, 20% methanol, pH 4.0) at 55˚C, for 5 h. Laura software v4.1.7.70 (LabLogic Systems Ltd) was used for the data analysis. The levels of peptides in crosslink (x) was calculated using the formula x = 100 - (%TetraTetra + %TetraPenta).

Metabolomics

Sampling of intracellular metabolites: for intracellular metabolite samples, 20 OD units of cells were harvested via vacuum-dependent fast-filtration system as described by Meyer *et al.*, (2014)[4] with modifications. In brief, the main culture was transferred into a falcon tube and cooled by dipping it periodically in liquid nitrogen for 10 s maximum (around 1 s each time). During this

in/out of liquid nitrogen cycle, the sample was carefully shaken to avoid freezing and metabolite leakage caused by cell lysis. The cooled cell culture was filtered (regenerated cellulose membrane filter, 0.45 µm pore size, 100 mm diameter, RC55 Whatman) and washed 2 times with isotonic 0.9% sodium chloride solution at 4ºC. The filter was immediately transferred to a falcon tube containing 5 mL of ice-cold extraction solution (60% w/v of ethanol absolute 99.8%) and internal standard (ISTD) constituted of 2.5 nmol of camphorsulphonic acid (CSA) for HPLC-MS and 20 nmol of ribitol for GC-MS analysis. The metabolites were quenched by freezing the sample immediately in liquid nitrogen. The falcon tube was stored at -80°C until extraction.

For cell disruption and metabolites extraction, 10 freeze/thaw cycle was performed by alternately thawing on ice, vortexing, and shaking the sample. Afterwards, the sample was centrifuged for 5 min at 4°C and 13000 *rpm*. The supernatant was collected to a new falcon tube and left on ice. A second extraction was carried out with 5 mL of deionized water. The two supernatants were combined and distilled water was added to get a final organic solution concentration of 10%. The sample was frozen and stored at -80°C. The sample was lyophilized with a Christ Alpha 1-4 LSC lyophilizer at -52°C and 0.25 mbar. The sample was stored at -20°C until analytical analyses.

GC-MS measurement and data analysis of intracellular metabolites. The dried samples were derivatized firstly with 60 µL of methoxyamine hydrochloride (20 mg/ml solution in pyridine) for 90 min at 37°C and secondly with 120 µL of N-methyl-N-trimethylsilyltrifluroacetamide (Chromatographie-Service GmbH) for 30 min at 37°C. Samples were centrifuged for 2 min at room temperature and the supernatant was transferred into GC-vial for injection. GC-MS analysis was performed with an Agilent 6890N GC system with an auto-sampler G2614A model coupled to a mass selective detector 5973N model (Agilent Technologies, USA). A 2 µL sample was injected (G2613A model series injector) with a split 1:10 at 250°C

using helium as the carrier gas (split flow of 10 mL/min and 8.8 Psi). The chromatographic run was performed as described by Dörries *et al.*, (2013)[5]. Using a 30 m DB 5-column (JW Scientific, Folsom, USA) with 0.25 mm inner diameter and 2.5 µm film thickness, and a constant gas flow of 1 mL/min^{-1}. The oven program started with an initial temperature hold at 70°C for 1 min and continued with a heating rate of 1ºC/min up to 76ºC, 5º C/min up to 220°C, and 20°C/min up to 330°C, with a hold for 3 min followed by a 10 min isothermal cool-down to 70°C. The analytes were transferred to a quadrupole mass analyser operated in the EI ionization mode with an ionization energy of 70 eV. Data acquisition was done in 40 min runtime. A full scan mass spectrum were acquired from 50 to 500 m/z at a rate of 2 scans/s and with a 6 min solvent delay. The qualitative analysis of the detected compounds was performed using ChromaTOF software (LECO Corporation, Michigan, USA). Metabolite identification was carried out by comparison of retention time and fragmentation patterns peaks detected to the NIST mass spectral database 2.0 (Gaithersburg, USA) and an in-house database. Identification of peaks was carried out by comparison of mass spectra with a database with a similarity of 75%. For relative quantification, the area of the quantifier ion of each metabolite was integrated and normalized to the area of the ISTD (ribitol). For precision analysis control, daily quality control (dQC) samples were analysed during the batch. The dQC consisted in 53 metabolites with 100 nmol concentration for each metabolite. Precision analysis was determined by assessing the measured dQC in calibration curves with concentrations ranging from 0.5 nmol to 500 nmol of each metabolite. The calibration curves fitting was performed with a polynomial of degree 2 and 1/x weighting based on minimum of 6 calibration points.

HPLC-MS measurement and data analysis of intracellular metabolites. The lyophilized samples were dissolved in 100 µL of water (HPLC-MS grade) and centrifuged for 2 min at room temperature. The supernatant was transferred into HPLC-vial for injection. HPLC-MS analysis

was carried out using an Agilent 1100 HPLC system consisted of a degasser, a quaternary pump and a G1329A autosampler with controlled temperature coupled to Bruker micro-TOF mass spectrometer (Bruker Daltonics, Bremen, Germany). Chromatography was performed on a SymmetryShield RP18 column (3.5 µm, 150 x 4.6 mm) (Waters, Milford, USA) with a SecurityGuard cartridge C18 pre-column (4 x 3.0 mm) (Phenomenex, Torrance, USA) using an ion-pairing reagent and a methanol gradient (4). In detail, the mobile phase consisted in eluent A: 95% water and 5% methanol, containing 10 mM of tributylamine as the ion-pairing reagent and 15 mM of acetic acid, pH 4.9; and eluent B: 100% methanol. Data acquisition was done in 42 min runtime with a flow rate of 0.4 mL/min. The gradient elution started with 100% A for 2 min, 0-31% B in 2 min and continued with 31 to 50% in 18 min followed by 50-60% B in 2 min, 60-100% B in 1 min and left 100% for 7 min. The eluent A returned to 100% in 1 min and was left for 10 min until the end of the run. The gradient elution started with 100% A for 2 min, 0-31% B in 2 min and continued with 31 to 50% in 18 min. Followed by 50-60% B in 2 min, 60-100% B in 1 min and left 100% for 7 min. The eluent A returned to 100% in 1 min and was left for 10 min until the end of the run.

Mass spectrometry was operated in electrospray ionization and negative-ion mode using a mass scan range of 50 to 3000 m/z. Internal MS calibration was carry out in the beginning of each chromatographic run with 16 different masses from a sodium formate solution tune mix (49.4% water, 49.4% isopropanol, 0.2% formic acid, and 10 mM sodium hydroxide). Metabolite identification was carried out by comparison of retention time and m/z values of detected peaks ($[M-H]^-$ or $[M-2H]^{2-}$) with database alignment of the calculated exact mass. The quantitative analysis was done using QuantAnalysis (Bruker Daltonik, Bremen, Germany). The extracted ion peaks were integrated and normalized to the ISTD (CSA) area. The dQC samples consisted in 22 metabolites with 10 nmol concentration of each metabolite. Precision analysis was determined by

assessing the measured dQC in calibration curves with concentrations ranging from 0.5 nmol to 500 nmol of each metabolite. The calibration curves fitting was performed with a polynomial of degree 2 and 1/x weighting based on minimum of 6 calibration points.

Statistical analysis and visualization: Microsoft Excel software 2007 was used for metabolites quantification and calculation of standard deviations (SD) and fold change (FC). Unpaired t-tests were also done in Excel. The two-sided homoscedastic t-tests were used to calculate p-values, whereas p-values$\leq$0.05 were considered statistically significant. Bar-charts and volcano-plots were generated using GraphPad PRISM software v6.01. The area of m/z detected for each metabolite was integrated and normalized to the integral of the area of m/z the internal standard, resulting in the relative metabolite amount per 20 OD units. The metabolite missing values were replaced with half the minimum positive value in the original data.

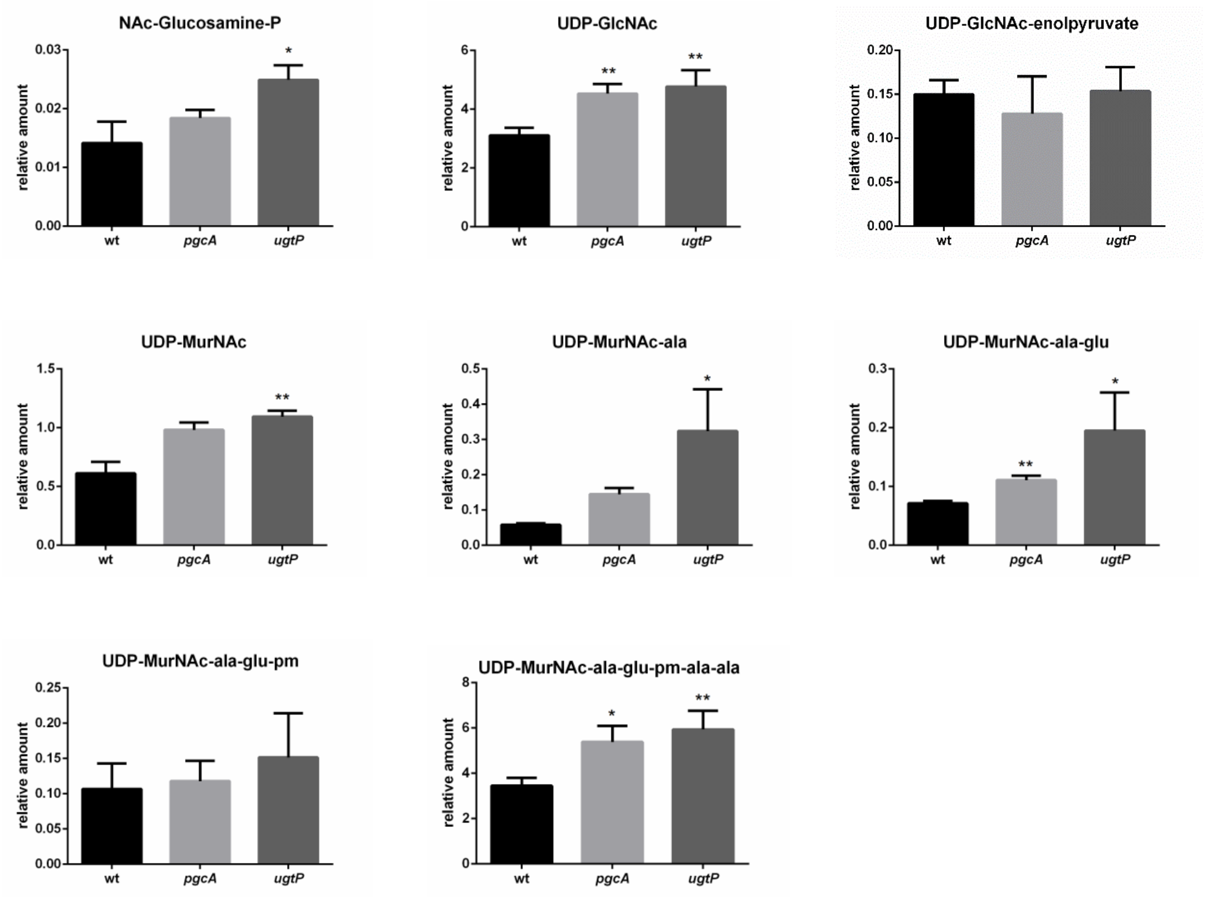

Fig. S1: Relative intracellular concentrations of peptidoglycan precursors in BSB1, *pgcA* and *ugtP* mutants.

Relative intracellular concentrations of peptidoglycan precursors in BSB1, *pgcA* and *ugtP* mutants in LB medium. Data are presented as mean values ± SD of biological three biological replicates. Statistical differences between control and mutants were considered significant for $p \leq 0.05$ (*) and $p \leq 0.01$ (**).

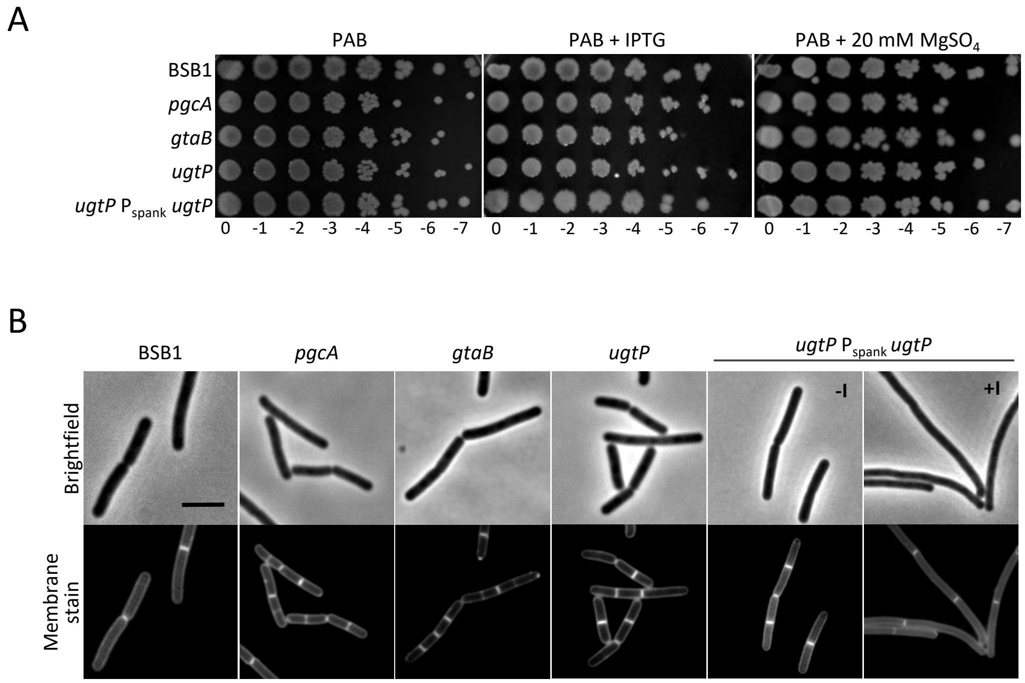

Fig. S2: the absence of UgtP, GtaB or PgcA resulted in short cells.

(**A**) Spot plate assay for mutants lacking UgtP, GtaB or PgcA. All mutant showed similar growth to wild type cells on PAB media. The ugtP complementation mutant also showed wild type growth. (**B**) Fluorescence microscopy using membrane stain showed short cells phenotype for the *ugtP*, *gtaB* or *pgcA* mutants. The ectopic expression of UgtP resulted in increase in cell length. -I, without IPTG; +I, with IPTG. Scale bar: 3 µm.

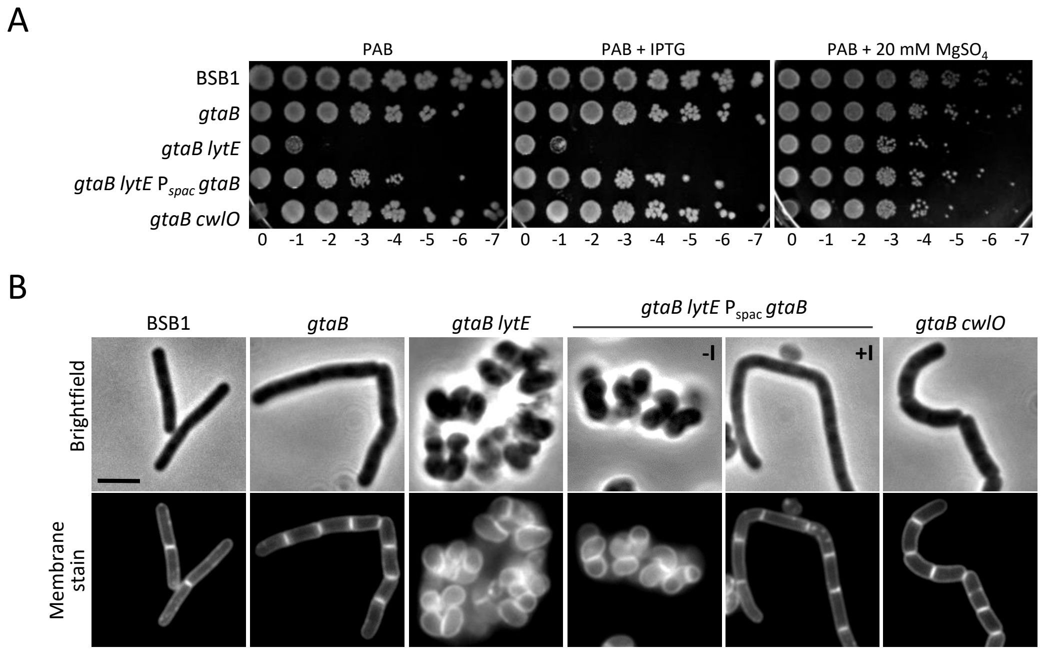

Fig. S3: The *gtaB* mutant requires LytE to maintain rod-shape.

(**A**) Spot plate assay showing the lethality of the *gtaB lytE* double knockouts when cells were grown on PAB media. This lethality was rescued by ectopically expressing GtaB or supplementing the plates with magnesium. The *gtaB cwlO* double mutant grew similarly to the *gtaB* single mutant. (**B**) Fluorescence microscopy showed a severe shape defect only for the *gtaB lytE* mutant when grown in LB media. The complementation of *gtaB* rescued this phenotype. -I, without IPTG; +I, with IPTG. Scale bar: 3 µm.

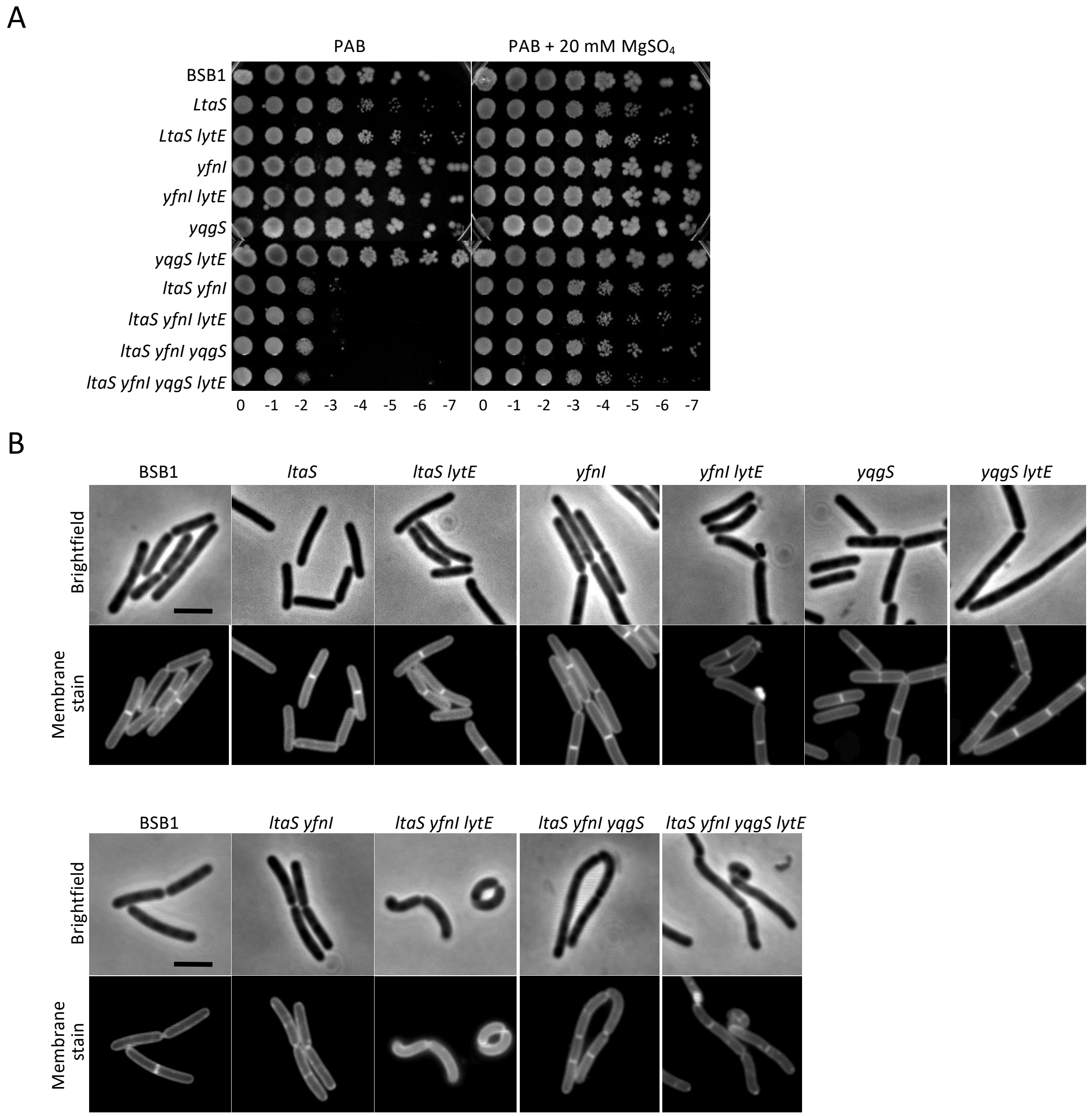

Fig. S4: LytE is dispensable for cells lacking LTA synthases.

(**A**) Spot plate assay for mutants lacking the LTA synthases LtaS, YfnI and/or YqgS. The BSB1 Δ*ltaS* Δ*yfnI* Δ*yqgS* triple mutant showed worse growth defect compared to single mutants or wild type cells. This latter phenotype was independent of the presence or absence of LytE. (**B**) Mutants were also analysed by fluorescence microscopy during exponential phase. BSB1 Δ*ltaS* Δ*yfnI* Δ*yqgS* exhibited morphological defects compared to single mutants of LTA synthases. BSB1 Δ*ltaS* Δ*yfnI* Δ*yqgS* Δ*lytE* showed similar morphology to BSB1 Δ*ltaS* Δ*yfnI* Δ*yqgS* with infrequent rod-shape defects. Scale bar: 3 µm.

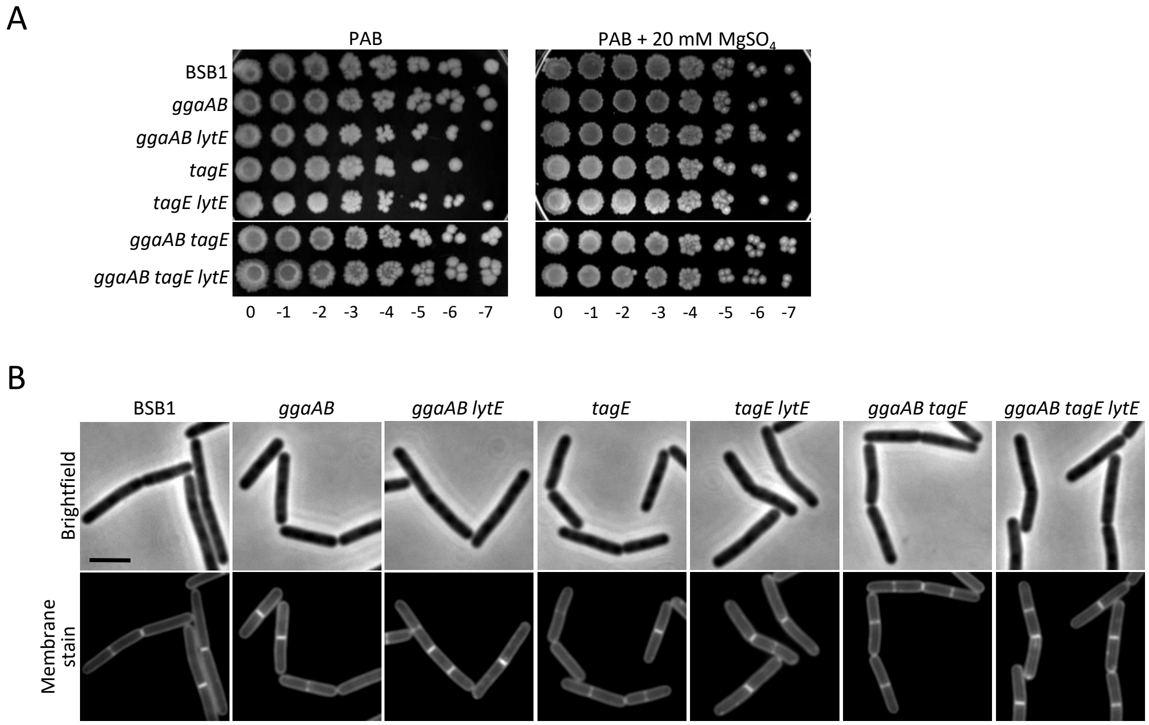

Fig S5: LytE is dispensable for cells lacking the glucose transferases TagE and GgaAB.

(**A**) Spot plate assay for mutants with defects in the glycosylation of the wall teichoic acid. BSB1 Δ*ggaAB* Δ*tagE* and BSB1 Δ*ggaAB* Δ*tagE* Δ*lytE* exhibited similar phenotype to wild type cells when grown on PAB. (**B**) Fluorescence microscopy for the aforementioned mutants showed comparable rod-shape to wild type cells. Scale bar: 3 μm.

A

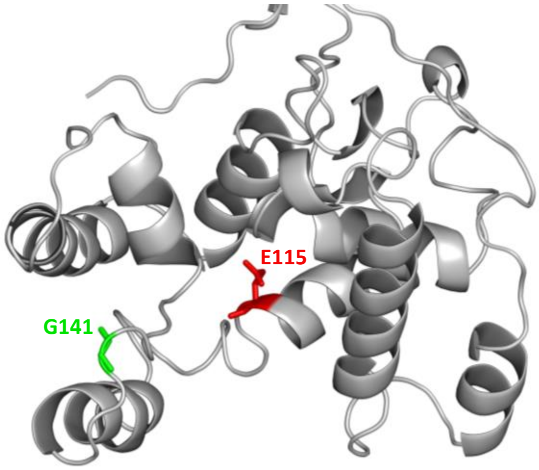

B

GTase catalytic residue (E115 residue)

ugtP lytE suppressor Mutation (G141 residue)

```
BsPBP1   KRTYVSIDEIPDVVKEAFIATEDARFYEHHGIDPVRIGGALVANFKDGFGAEGGSTITQQVVKNSLLSHQKTLKRKVQEVWLSIQL 179
SaPBP2   RHEHVNLKDVPKSMKDAVLATEDNRFYEHGALDYKRLFGAIGKNLTGGFGSEGASTLTQQVVKDAFLSQHKSIGRKAQEAYLSYRL 178
         :: :*.:.::*. :*:*.:**** *****  .:*   *: **:   *:..***:**.**:******::**::*:: **.**.:** :*
```

Fig. S6: Ribbon representation of the GTase domain structure of PBP1 modelled to PBP2 from *S. aureus* (SaPBP2B) using Phyre[2].

(**A**) Ribbon representation of the GTase domain of BsPBP1 and highlighting the glutamate active site E115 in red and the suppressor mutation G141 in green (Fig. 6). (**B**) Sequence alignment of the GTase domains of the two proteins showing high levels of resemblance between the sequences of the two PBPs.

Table S1. List of strains and plasmids.

Strain/ Plasmid	Characteristics	Reference/Source
Strain		
168CA	*TrpC2*	Laboratory collection
PG237	*TrpC2* Δ*ugtP*::*neo*	Laboratory collection
SSB122	*TrpC2* Δ*pgcA*::*tet*	[6]
JS07	*TrpC2* Δ*gtaB*::*erm*	This work
PDC463	*TrpC2* Δ*cwlO*::*spc*	[7]
PDC464	*TrpC2* Δ*lytE*::*cat*	[7]
PS2062	*TrpC2* Δ*ponA*::*spc*	[8]
BGSC3	*TrpC2* Δ*ponA*::*erm*	BGSC
BSB1	Autotroph	[9]
JS03	BSB1 Δ*ugtP*::*neo*	BSB1 transformed with PG237 DNA
JS05	BSB1 Δ*ugtP*::*neo amyE*::(P_{spank} *ugtP spc*)	This work
JS04	BSB1 Δ*pgcA*::*tet*	This work
JS09	BSB1 Δ*gtaB*::*erm*	This work
JS12	BSB1 Δ*gtaB*::*erm aprE*::(P_{spac} *gtaB spc*)	This work
JS165	BSB1 Δ*gtaB*::erm Δ*lytE*::*cat*	This work
JS166	BSB1 Δ*gtaB*::*erm* Δ*lytE*::*cat aprE*::(P_{spac} *gtaB spc*)	This work
JS167	BSB1 Δ*gtaB*::*erm* Δ*cwlO*::*spc*	This work
JS06	BSB1 Δ*ponA*::*cat*	This work
JS42	BSB1 Δ*cwlO*::*spc*	This work
JS43	BSB1 Δ*lytE*::*cat*	This work
JS13	BSB1 Δ*ugtP*::*neo* Δ*cwlO*::*spc*	This work
JS44	BSB1 Δ*ugtP*::*neo* Δ*lytE*::*cat*	This work

JS15	BSB1 Δ*ugtP*::*neo* Δ*ponA*::*cat* *amyE*::(P_{spank} *ugtP spc*)	This work
JS14	BSB1 Δ*ugtP*::*neo* Δ*lytE*::*cat* *amyE*::(P_{spank} *ugtP spc*)	This work
PDC639	*TrpC2* Δ*cwlO*::*spc* *aprE*::(P_{xyl} *cwlO erm*)	(Dominguez-Cuevas *et al*., 2013)
JS77	BSB1 Δ*ugtP*::*neo* *amyE*::(P_{xyl} *cwlO erm*)	This work
JS78	BSB1 Δ*ugtP*::*neo* Δ*lytE*::*cat* *amyE*::(P_{xyl} *cwlO erm*)	This work
4283	trpC2 Δ*ltaS*::*neo*	[10]
4285	trpC2 Δ*ltaS*::*cat*	[10]
JS94	BSB1 Δ*ltaS*::*neo*	This work
JS96	BSB1 Δ*ugtP*::*neo* Δ*ltaS*::*cat*	This work
4288	trpC2 Δ*yfnI*::*cat*	[10]
4289	trpC2 Δ*yfnI*::*erm*	[10]
JS115	BSB1 Δ*yfnI*::*cat*	This work
JS117	BSB1 Δ*yfnI*::*erm* Δ*lytE*::*cat*	This work
4291	*trpC2* Δ*yqgS*::*neo*	[10]
4293	*trpC2* Δ*yqgS*::*cat*	[10]
JS111	BSB1 Δ*yqgS*::*cat*	This work
JS113	BSB1 Δ*yqgS*::*neo* Δ*lytE*::*cat*	This work
JS141	BSB1 Δ*ltaS*::*neo* Δ*yfnI*::*erm*	This work
JS142	BSB1 Δ*ltaS*::*neo* Δ*yfnI*::*erm* Δ*lytE*::*cat*	This work
JS151	BSB1 Δ*ltaS*::*neo* Δ*yfnI*::*erm* Δ*yqgS*::*cat*	This work
PDC687	*trpC2* Δ*lytE*::*cat*::*spc*	[7]
JS157	BSB1 Δ*ltaS*::*neo* Δ*yfnI*::*erm* Δ*yqgS*::*cat* Δ*lytE*::*cam*::*spc*	This work
MH1024	168 Δ*ggaAB*::*spc*	[11]
JS178	BSB1 Δ*ggaAB*::*spc*	This work
JS171	BSB1 Δ*lytE*::*cat* Δ*ggaAB*::*spc*	This work

SM50	*trpC2* Δ*tagE*::*erm*	Richard Daniel strain collection
JS182	BSB1 Δ*tagE*::*erm*	This work
JS172	BSB1 Δ*lytE*::*cat tagE*::*erm*	This work
JS174	BSB1 Δ*tagE*::*erm* Δ*ggaAB*::*spc* Δ*lytE*::*cat*	This work
JS79	BSB1 Δ*lytE*::*cat ugtP*::*neo ponA*::*erm*	This work
JS185	BSB1 Δ*ugtP*::*neo* Δ*lytE*::*cat* Δ*ponA*::*erm aprE*:: (P_{spac} *ponA spc*)	This work
JS186	BSB1 Δ*ugtP*::*neo* Δ*lytE*::*cat* Δ*ponA*::*erm aprE*:: (P_{spac} *ponA* E115A *spc*)	This work
JS187	BSB1 Δ*ugtP*::*neo* Δ*lytE*::*cat* Δ*ponA*::*erm aprE*:: (P_{spac} *ponA* G141A *spc*)	This work
4261	*trpC2* Δ*mbl*::*cat*	[12]
4281	*trpC2* Δ*mreB*::*cat*	[12]
PDC627	*trpC2* Δ*mreBH*::*spc*	[7]
JS125	BSB1 Δ*mbl*::*cat*	This work
JS126	BSB1 Δ*mreB*::*cat*	This work
JS127	BSB1 Δ*mreBH*::*erm*	This work
JS128	BSB1 Δ*mbl*::*cat* Δ*ugtP*::*neo*	This work
JS129	BSB1 Δ*mreB*::*cat* Δ*ugtP*::*neo*	This work
JS130	BSB1 Δ*mreBH*::*erm* Δ*ugtP*::*neo*	This work
Plasmid		
pDR111	*Bla amyE*::(*spc lacI* P_{spank})	David Rudner
pAPNC213	*Bla aprE*::(*spc lacI* P_{spac})	[13]
pJS001	*Bla amyE*::(*spc lacI* P_{spank} *ugtP*)	This work
pJS003	*Bla aprE*::(*spc lacI* P_{spank} *ugtP*)	This work
PcotC-GFP	*Bla cat* P_{CotC} *cotC-gfp*	[14]

Table S2. List of nucleotides.

Name	Restriction site	5'-3' sequence	Reference/comment
AG124	-	CCATCATCTGGTGCGAAAGG	5' *ponA* for sequencing
AG125	-	CCGCAAAGCCGATTAATTGG	3' *ponA* for sequencing
JS01	-	AAGCACACGCAGGTCATTTG	Check for integration into *aprE*
JS02	-	CCATCCGTCGATCATGGAAC	Check for integration into *aprE*
JS05	SalI	CGGTCGACGCTTGTTGTTGATTACATTGAGGTG	5' *ugtP* (construction of pJS01)
JS06	SphI	CTGCATGCCGTATGCTCTCAAGTACGCC	3' *ugtP* (construction of pJS01)
JS09	SalI	GAGTCGACCGATCATAAGGAAGGTGC	5' *gtaB* (construction of pJS03)
JS10	EcoRI	GTGAATTCGCCGTTGATCAGGTCTTCGCAG	3' *gtaB* (construction of pJS03)
JS17	-	GTGGCGACAGATTACGTGAAGG	5' *ugtP* for sequencing
JS18	-	TTGCTTGGATGAGTGCCGATCTCCAG	3' *ugtP* for sequencing
JS19	-	CTCCACTGTTACATCGCCGAACC	5' *pgcA* for sequencing
JS20	-	TCGCGTTTACCTGCTCAATGAC	3' *pgcA* for sequencing
JS35	-	TCCGTTTCCCGCATCTCAGCCTC	*5' ponA* upstream (construction of JS06)
JS36	-	CCGTTCCCAAGACTGTTAAACC	*3' ponA* downstream (construction of JS06)
JS43		GAAAGCGCCCTTTCCGATATTAC	*5' gtaB* upstream (construction of JS07)
JS46		CCTCTTCCAAAGTAATATCGACACATGC	*3' gtaB* downstream (construction of JS07)
JS49	-	ACCACCAGTGATTATGCC	Check for integration in *amyE* (5')
JS50	-	CCGCTCGCCATGACTTCACTAAC	Check for integration in *amyE* (3')
JS51	XbaI	TCTAGAACGCTAGCACCCATTAGTTCAACAAACG	Amplification of *Cm* cassette from P_{CotC}-*cotC-gfp* (5')
JS53	BsgI	CGTGCAGAATTCGTACAGTCGGCATTATCTC	Amplification of *Cm* cassette from P_{CotC}-*cotC-gfp* (3')
JS56	-	GCTCTAGATTTCGGTAATCAGCTCATCAAG	*5' ponA* downstream (construction of JS06)
JS57	XbaI	GCTCTAGATCACGGCTGTTAAATTGATCTG	*3' ponA* upstream (construction of JS06)

JS67	XbaI	CGCTCTAGAAAGACGGTTCGTGTTCGTGCTGAC	Amplification of *erm* cassette from pMUTIN4 (5’)
JS68	EcoRI	CGCGAATTCAGCTCCTTGGAAGCTGTCAGTAG	Amplification of *erm* cassette from pMUTIN4 (3’)
JS69	XbaI	CGCTCTAGAAGCCTGCTGCTGGAATTATGGCTTTACG	*3’ gtaB* upstream (construction of JS07)
JS70	EcoRI	CAGGAATTCGCTCTTCATTATCAACTGCGAAGAC	*5’ gtaB* downstream (construction of JS07)
JS82	-	CCCTACAGTGTTATGGCTTGAACAATC	5’ $P_{hy\text{-}spank}$ for pDR111
JS83	-	CCCTACAGTGTTATGGCTTGAACAATC	3’ *amyE* for pDR111
JS88	-	GTCTGTGCTTGAGGATAAGG	*lytE* upstream
JS89	-	GATCCGTTTGCGTGTTTC	*lytE* downstream
JS90	-	CCCGCTCCCGACATTCCAGTTATAATGAC	*cwlO* upstream
JS91	-	GTTAATGGCTTCCCATGGCCTTTACC	*cwlO* downstream
JS93	-	GGTGATTGTAATGAAGCTCAG	*3’ ugtP* downstream
AG316		CGCCACTTTCTCCCTCATAC	5’ *ltaS* for sequencing
AG317		GTCAAATCGGGCGGGCAATC	3’ *ltaS* for sequencing
KS1	-	GCTTTAAGAAAGGAAGATACAT	5’ *mreB* for sequencing
KS2	-	TCGACAATTGTAGATACAGT	3’ *mreB* for sequencing
KS3	-	TTAAATCTGTAAGGTCAGCC	3’ *mreB* for sequencing
KS4	-	AAATTAGGATAGAGATTGGGT	5’ *mreBH* for sequencing
KS5	-	TGGCTCTTCGATTAAATGAA	3’ *mreBH* for sequencing
KS6	-	TCCATTTCCCACAATATGAA	3’ *mreBH* for sequencing
KS7	-	GGATATTTACTGTGAAACAGAT	5’ *mbl* for sequencing
KS8	-	AGAAGAGGAGGTGACAATAT	3’ *mbl* for sequencing
KS9	-	CCTATTATCGTCATTTAACATCT	3’ *mbl* for sequencing

Table S3: Relative levels of intracellular metabolomics.

	Fold change (mutant/wild type)		$\log_2$ Fold change		p-value	
Metabolite	Δ*pgcA*	Δ*ugtP*	Δ*pgcA*	Δ*ugtP*	Δ*pgcA*	Δ*ugtP*
2-oxoglutarate	1.169	1.116	0.226	0.159	0.370	0.354
2-phosphoglycerate	6.561	2.721	2.714	1.444	0.023	0.360
3-phosphoglycerate	2.806	1.466	1.489	0.552	0.019	0.194
5-methyluridine	1.224	1.097	0.292	0.134	0.032	0.231
5-oxoproline	1.079	1.093	0.110	0.128	0.308	0.521
Acetyl adenylate	1.212	1.045	0.277	0.064	0.079	0.505
Acetyl-CoA	1.663	1.321	0.734	0.402	0.016	0.506
Adenine	1.748	1.423	0.806	0.509	0.001	0.316
Adenosine	0.928	1.159	-0.107	0.213	0.621	0.227
Adenylsuccinate	0.997	0.520	-0.004	-0.943	0.981	0.011
ADP	0.688	0.966	-0.539	-0.050	0.089	0.853
AICAR	1.267	1.109	0.341	0.149	0.354	0.537
AMP	0.575	1.405	-0.799	0.491	0.220	0.182
Asparagine	0.909	1.500	-0.138	0.585	0.337	0.016
Aspartate	1.410	1.192	0.496	0.253	0.005	0.319
ATP	1.566	1.112	0.647	0.153	0.001	0.679
CAIR	1.203	1.111	0.267	0.152	0.533	0.578
cCMP	1.124	1.017	0.168	0.025	0.394	0.895
cdiAMP	1.238	1.008	0.308	0.012	0.018	0.923
CDP	0.549	1.156	-0.865	0.209	0.003	0.459
CDP-glucose	0.108	0.658	-3.217	-0.604	0.004	0.547
CDP-glycerol	9.189	3.924	3.200	1.972	0.002	0.151
Citrate	1.119	1.175	0.162	0.233	0.386	0.026
CMP	0.724	0.640	-0.466	-0.643	0.361	0.252
CoA	0.854	0.892	-0.228	-0.165	0.519	0.377
CTP	1.151	1.890	0.203	0.919	0.340	0.145
Cysteine	1.343	1.225	0.426	0.293	0.051	0.298
Cytidine	1.436	1.163	0.522	0.218	0.246	0.611
dADP	0.530	0.947	-0.915	-0.078	0.026	0.805
D-Ala-D-Ala	1.984	0.727	0.988	-0.460	0.012	0.391
D-Alanine	1.353	1.327	0.436	0.409	0.541	0.594
dATP	1.257	1.047	0.330	0.066	0.097	0.902
dCDP	0.565	0.623	-0.823	-0.683	0.006	0.011
dCMP	1.187	1.042	0.248	0.060	0.210	0.714
dCTP	1.441	1.499	0.527	0.584	0.012	0.130
Deoxycytidine	1.122	0.922	0.166	-0.117	0.261	0.376
Deoxythymidine	1.069	1.124	0.096	0.169	0.161	0.053
Deoxyuridine	1.113	1.117	0.155	0.159	0.345	0.346

dGDP	0.698	0.966	-0.518	-0.051	0.099	0.852
dGMP	0.569	1.433	-0.815	0.519	0.219	0.169
Dihydroxyacetone P	11.038	2.213	3.464	1.146	0.008	0.639
dTDP	0.579	1.118	-0.789	0.161	0.059	0.360
dTTP	1.598	1.289	0.676	0.367	0.004	0.158
Erythrose 4-P	1.015	0.879	0.022	-0.186	0.951	0.623
FAD	0.647	0.912	-0.629	-0.134	0.012	0.546
FGAR	1.000	1.516	0.001	0.600	0.999	0.200
Fructose 1,6-bisP	2.936	1.211	1.554	0.277	0.006	0.516
Fructose 6-P	1795	665	10.810	9.377	0.111	0.249
Fumarate	1.835	1.403	0.876	0.489	0.000	0.058
GAR	0.775	0.833	-0.368	-0.263	0.021	0.160
GDP	0.510	0.717	-0.973	-0.480	0.058	0.163
Gluconate	1.013	1.168	0.018	0.224	0.888	0.576
GluconateP	1.087	1.086	0.121	0.119	0.259	0.142
Glucose	0.842	1.004	-0.249	0.006	0.031	0.903
Glucose 6-P	796	229	9.637	7.841	0.015	0.221
Glucuronate	6.870	0.037	2.780	-4.758	0.042	0.374
Glutamate	1.106	1.424	0.146	0.510	0.833	0.029
Glutamine	22.77	8.65	4.509	3.113	0.001	0.011
Glycerate	1.263	1.130	0.337	0.176	0.051	0.020
Glycine	1.098	1.040	0.135	0.056	0.293	0.850
GMP	1.200	1.105	0.263	0.144	0.035	0.136
GTP	1.456	0.930	0.542	-0.105	0.012	0.823
Guanine	1.344	0.882	0.427	-0.182	0.013	0.323
Guanosine	1.161	1.508	0.215	0.593	0.593	0.259
Histidine	8.699	4.911	3.121	2.296	0.001	0.137
Hypoxanthine	1.038	1.072	0.054	0.100	0.828	0.644
IMP	0.571	1.331	-0.809	0.412	0.182	0.204
Isoleucine	0.905	0.919	-0.144	-0.121	0.526	0.686
ITP	1.584	1.159	0.664	0.212	0.001	0.554
Lactate	1.258	1.211	0.331	0.276	0.019	0.068
Leucine	1.055	0.957	0.077	-0.063	0.750	0.877
Lysine	1.002	1.089	0.003	0.123	0.982	0.657
Malate	1.578	0.957	0.658	-0.063	0.010	0.630
Malonyl-CoA	4.674	2.139	2.225	1.097	0.001	0.364
Methionine	1.126	1.057	0.171	0.081	0.194	0.710
Myo-inositol	1.396	1.196	0.481	0.258	0.038	0.546
NAcGlucosamine-P	1.304	1.763	0.383	0.818	0.129	0.013
NAD	1.191	1.191	0.252	0.252	0.227	0.388
NADH	1.089	1.199	0.122	0.262	0.541	0.390
NADP	1.274	1.079	0.349	0.109	0.231	0.802
NADPH	1.156	1.201	0.209	0.264	0.417	0.588

Ornithine	1.262	1.170	0.336	0.227	0.022	0.391
Phosphoenolpyruvate	4.085	2.021	2.030	1.015	0.029	0.165
PRA	1.096	1.197	0.132	0.259	0.473	0.168
Proline	0.913	0.864	-0.131	-0.211	0.605	0.599
PRPP	1.165	1.308	0.220	0.387	0.366	0.400
Pseudouridine	1.126	1.026	0.171	0.037	0.176	0.748
Pyruvate	0.570	0.722	-0.811	-0.469	0.035	0.086
Ribose 1,5 bisP	0.787	1.029	-0.345	0.041	0.327	0.750
SAICAR	1.247	0.976	0.318	-0.034	0.050	0.773
Sedoheptulose 1,7-bisP	1.863	0.956	0.898	-0.065	0.046	0.854
Sedoheptulose 7-P	1.032	1.417	0.046	0.503	0.908	0.099
Serine	1.104	1.057	0.142	0.080	0.229	0.725
Succinate	1.066	1.254	0.092	0.326	0.475	0.063
SuccinylCoA	1.131	0.605	0.178	-0.725	0.776	0.185
Threonine	1.096	1.052	0.132	0.073	0.293	0.742
Trehalose 6-P	0.716	1.065	-0.482	0.091	0.370	0.877
Tyrosine	1.078	1.082	0.109	0.114	0.380	0.611
UDP	0.586	1.047	-0.772	0.066	0.042	0.729
UDP-GlcA	0.001	0.485	-10.740	-1.044	0.000	0.011
UDP-GlcNAc	1.460	1.536	0.546	0.619	0.004	0.009
UDP-GlcNAc-enolpyruvate	0.854	1.026	-0.228	0.037	0.453	0.845
UDP-Glucose	0.000	0.994	-11.919	-0.008	0.000	0.985
UDP-MurNAc	1.606	1.790	0.684	0.840	0.005	0.002
UDP-MurNAc-5aa	1.562	1.721	0.643	0.784	0.013	0.009
UDP-MurNAc-ala	2.500	5.588	1.322	2.482	0.001	0.018
UDP-MurNAc-ala-glu	1.554	2.734	0.636	1.451	0.001	0.030
UDP-MurNAc-ala-glu-pm	1.078	1.472	0.109	0.557	0.780	0.296
UMP	0.686	1.341	-0.545	0.423	0.042	0.033
Uracil	0.976	1.020	-0.036	0.028	0.730	0.921
Uridine	1.044	1.039	0.062	0.055	0.647	0.621
UTP	1.814	1.508	0.859	0.593	0.001	0.158
Valine	1.339	1.158	0.421	0.212	0.004	0.641
XMP	0.641	0.828	-0.642	-0.272	0.000	0.017
XTP	1.445	0.941	0.531	-0.088	0.023	0.848

Table S4. Cell size measurements and morphology.

Strain, with or without inducer IPTG	Cell length (µm)[1]	Cell width (µm)[1]	bent cells (%)[1]
BSB1	3.6 ± 0.5	1.2 ± 0.10	0
BSB1 Δ*ugtP*	2.0 ± 0.3	1.3 ± 0.05	0
BSB1 Δ*gtaB*	2.3 ± 0.5	1.3 ± 0.05	0
BSB1 Δ*pgcA*	2.5 ± 0.6	1.3 ± 0.06	0
BSB1 Δ*lytE*	3.0 ± 0.6	1.1 ± 0.07	20
BSB1 Δ*cwlO*	3.0 ± 0.7	1.2 ± 0.06	0
BSB1 Δ*ugtP* Δ*cwlO*	2.5 ± 0.6	1.4 ± 0.08	0
BSB1 Δ*ugtP* Δ*lytE*	N.A	N.A	>90
BSB1 Δ*ugtP* Δ*lytE* P_{spank} *ugtP*, no IPTG	3.1 ± 0.8	1.3 ± 0.08	20
BSB1 Δ*ugtP* Δ*lytE* P_{xyl} *cwlO*, no Xylose	2.8 ± 0.6	1.3 ± 0.06	25
BSB1 Δ*ugtP* Δ*lytE* P_{xyl} *cwlO*, 0.5% Xylose	2.7 ± 0.7	1.3 ± 0.05	10
BSB1 Δ*ltaS*	3.4 ± 0.7	1.0 ± 0.04	0
BSB1 Δ*ltaS* Δ*lytE*	3.1 ± 0.8	1.1 ± 0.05	16
BSB1 Δ*yfnI*	3.9 ± 0.9	1.1 ± 0.05	0
BSB1 Δ*yfnI* Δ*lytE*	3.3 ± 0.7	1.1 ± 0.05	0
BSB1 Δ*yqgS*	3.3 ± 0.8	1.0 ± 0.03	0
BSB1 Δ*yqgS* Δ*lytE*	3.1 ± 0.7	1.1 ± 0.07	0
BSB1 Δ*ltaS* Δ*yfnI*	3.4 ± 0.9	1.0 ± 0.05	10
BSB1 Δ*ltaS* Δ*yfnI* Δ*lytE*	3.2 ± 0.9	1.1 ± 0.08	40
BSB1 Δ*ltaS* Δ*yfnI* Δ*yqgS*	4.0 ± 0.9	1.0 ± 0.04	30
BSB1 Δ*ltaS* Δ*yfnI* Δ*yqgS* Δ*lytE*	3.5 ± 0.9	1.0 ± 0.05	55
† BSB1 Δ*mbl*	3.3 ± 0.8	1.3 ± 0.10	0
† BSB1 Δ*mbl* Δ*lytE*	2.8 ± 0.7	1.5 ± 0.10	0
† BSB1 Δ*mreBH*	3.0 ± 0.6	1.1 ± 0.04	0
† BSB1 Δ*mreBH* Δ*lytE*	2.6 ± 0.6	1.3 ± 0.07	0
† BSB1 Δ*mreB*	2.7 ± 0.6	1.4 ± 0.10	0
† BSB1 Δ*mreB* Δ*lytE*	2.0 ± 0.6	1.7 ± 0.20	N/A
BSB1 Δ*ugtP* Δ*lytE* Δ*ponA*	3.2 ± 0.8	0.8 ± 0.04	52
BSB1 Δ*ugtP* Δ*lytE* Δ*ponA* P_{spank} *ponA*, no IPTG	2.9 ± 1.0	0.9 ± 0.06	55
BSB1 Δ*ugtP* Δ*lytE* Δ*ponA* P_{spank} *ponA*, 0.1 mM IPTG	2.8 ± 0.7	1.2 ± 0.07	5
BSB1 Δ*ugtP* Δ*lytE* Δ*ponA* P_{spank} *ponA*, 0.5 mM IPTG	2.3 ± 0.5	1.3 ± 0.09	8
BSB1 Δ*ugtP* Δ*lytE* Δ*ponA* P_{spank} *ponA* E115A, no IPTG	3.5 ± 1.0	0.8 ± 0.05	48
BSB1 Δ*ugtP* Δ*lytE* Δ*ponA* P_{spank} *ponA* E115A, 0.1 mM IPTG	2.6 ± 1.0	0.8 ± 0.05	55
BSB1 Δ*ugtP* Δ*lytE* Δ*ponA* P_{spank} *ponA* E115A, 0.5 mM IPTG	2.7 ± 0.9	0.8 ± 0.06	66
BSB1 Δ*ugtP* Δ*lytE* Δ*ponA* P_{spank} *ponA* G141A, no IPTG	3.1 ± 0.9	0.8 ± 0.04	56
BSB1 Δ*ugtP* Δ*lytE* Δ*ponA* P_{spank} *ponA* G141A, 0.1 mM IPTG	3.1 ± 0.7	1.1 ± 0.07	0
BSB1 Δ*ugtP* Δ*lytE* Δ*ponA* P_{spank} *ponA* G141A, 0.5 mM IPTG	2.7 ± 0.6	1.1 ± 0.07	0

[1] values are mean ± SD of >100 cells.

Cells were grown in LB media, except if labelled with † where LB + 20 mM $MgSO_4$ was used as an alternative growth media. All measurements are significant compared to BSB1.

Table S5. Muropeptide composition of strains.

Muropeptides	Peak no	Peak area (%)[1]					
		BSB1	BSB1 Δ*ugtP*	BSB1 Δ*pgcA*	BSB1 Δ*lytE*	BSB1 Δ*ugtP* Δ*lytE*	BSB1 Δ*ugtP* Δ*cwlO*
Tri	1	2.2±0.0	1.9±0.0	1.8±0.1	2.7±0.1	2.0±0.1	2.0±0.0
Tri (NH_2) (PO_4)	2	1.2±0.1	0.7±0.0	1.0±0.3	1.2±0.0	1.1±0.1	1.4±0.0
Tri (NH_2)	3	14.5±1.8	11.8±0.3	12.1±0.9	15.7±0.6	13.4±2.0	12.2±0.0
Tri (NH_2) (deAc)	4	0.1±0.0	0.1±0.0	0.1±0.0	0.1±0.0	0.1±0.0	0.1±0.0
Di	5	2.3±0.1	4.0±0.0	3.2±0.2	2.1±0.0	3.5±1.2	2.7±0.1
Tri-Ala-mDap (NH_2)	6	1.2±0.2	1.5±0.0	1.2±0.2	1.1±0.1	0.7±0.3	1.2±0.0
tetra (NH_2)	7	0.5±0.0	0.3±0.0	0.2±0.0	0.5±0.0	0.9±0.1	0.4±0.0
Tri-Ala-mDap $(NH_2)_2$	8	1.5±0.0	2.9±0.0	2.2±0.3	1.5±0.1	2.6±1.2	2.6±0.1
Penta (Gly5) (NH_2)	9	0.3±0.0	0.3±0.0	0.2±0.0	0.3±0.0	0.3±0.0	0.4±0.0
TetraTri (-GM) $(NH_2)_2$	10	0.7±0.0	0.6±0.0	0.7±0.3	0.7±0.0	0.9±0.0	1.5±0.0
Penta (NH_2)	11	0.4±0.0	0.6±0.0	0.6±0.2	0.4±0.0	0.5±0.0	0.5±0.0
TetraTri (-G)	12	0.8±0.1	0.4±0.0	0.4±0.1	0.7±0.0	0.5±0.1	0.4±0.0
TetraTri (NH_2) (PO_4)	13	0.8±0.2	1.5±0.3	1.6±0.7	0.7±0.1	0.7±0.1	0.7±0.1
TetraTetra (-GM) $(NH_2)_2$	14	1.0±0.2	0.6±0.0	0.7±0.0	0.6±0.3	0.6±0.3	0.5±0.1
TetraTri (NH_2)	15	15.5±3.7	10.9±8.9	11.3±3.0	17.9±0.1	10.5±42.3	10.9±0.1
TetraTri (NH_2) (deAc)	18	2.3±0.3	1.1±0.1	1.1±0.5	1.9±0.5	3.5±4.0	1.1±0.1
TetraTri (NH_2) (deAc)	19	1.7±0.3	0.8±0.0	0.9±0.4	1.3±0.3	0.6±0.1	0.7±0.0
TetraTri (NH_2)	20	3.2±0.2	2.8±0.0	2.7±0.1	4.3±0.3	4.2±0.1	4.6±0.6
TetraTri $(NH_2)_2$	21	25.7±0.0	28.3±0.8	29.2±2.2	27.6±3.2	33.5±4.5	31.1±0.9
TetraTri $(NH_2)_2$ (deAc)	22	1.0±0.0	0.1±0.0	0.1±0.1	1.5±0.2	0.2±0.0	2.0±0.0
TetraTri $(NH_2)_2$ (deAc)	23	0.4±0.0	0.3±0.0	0.2±0.0	0.5±0.1	0.6±0.0	0.3±0.0
Penta (Gly5) Tetra	24	0.3±0.0	0.6±0.0	0.5±0.1	0.1±0.0	0.1±0.0	0.3±0.0
Penta (Gly5) Tetra $(NH_2)_2$	25	0.5±0.1	0.7±0.0	0.6±0.0	0.4±0.0	0.5±0.0	0.6±0.1
TetraTetra $(NH_2)_2$	26	0.5±0.0	1.4±0.0	1.3±0.2	0.5±0.0	0.7±0.0	0.9±0.0
PentaTetra $(NH_2)_2$	27	0.5±0.0	0.5±0.0	0.4±0.1	0.5±0.0	0.7±0.0	0.7±0.0
TetraTetraTri $(NH_2)_2$	28	0.3±0.0	0.4±0.0	0.3±0.1	0.2±0.1	0.5±0.0	0.6±0.1
TetraTetraTri (-G)	29	1.2±0.0	1.0±0.1	1.0±0.3	0.8±0.0	0.6±0.0	0.7±0.0
TetraTetraTri $(NH_2)_2$	30	0.6±0.0	0.7±0.0	0.7±0.1	0.5±0.0	0.4±0.0	0.6±0.0
TetraTetraTri $(NH_2)_3$	31	3.1±0.2	2.5±0.4	2.3±0.6	2.9±0.1	2.4±0.0	2.5±0.0
TetraTetraTri $(NH_2)_3$ (deAc)	32	2.9±0.0	3.3±0.0	3.3±0.4	2.9±0.3	3.7±1.1	4.0±0.0
TetraTetraTri $(NH_2)_3$ (deAc)	33	0.6±0.0	0.6±0.0	0.6±0.1	0.4±0.0	0.3±0.0	0.7±0.0
Penta(Gly5)TetraTetra $(NH_2)_{2\text{-}3}$	34	0.3±0.0	0.3±0.0	0.3±0.0	0.2±0.0	0.2±0.0	0.3±0.0
TetraTetraTetraTri $(NH_2)_{2\text{-}3}$	35	0.5±0.0	0.4±0.0	0.3±0.0	0.7±0.0	0.4±0.0	0.3±0.0
TetraTri(Anh) $(NH_2)_2$	36	0.6±0.0	0.8±0.1	0.7±0.3	0.5±0.1	0.8±0.4	0.9±0.2
TetraTetraTetraTri $(NH_2)_4$	37	0.8±0.0	1.4±0.0	1.5±0.2	0.5±0.1	0.7±0.2	0.8±0.4
TetraTetraTri(Anh) $(NH_2)_2$	38	0.1±0.0	0.0±0.0	0.0±0.0	0.1±0.0	0.1±0.0	0.2±0.0
Sum monomers		24.0±0.1	24.8±0.7	23.9±0.3	25.6±1.6	24.3±0.3	23.0±0.2
Sum dimers		61.3±0.1	59.6±1.2	61.4±0.8	63.2±0.4	63.0±0.7	62.6±0.0
Sum trimers		11.4±0.0	10.2±0.0	9.9±0.2	8.4±0.5	8.6±0.5	10.4±0.3
Sum tetramers		2.2±0.0	2.1±0.0	2.1±0.3	1.4±0.1	1.3±0.4	1.3±0.3
Sum dipeptides		2.3±0.0	4.7±0.2	3.8±0.3	2.2±0.0	3.8±1.6	3.0±0.2
Sum tripeptides		55.7±0.1	53.9±0.2	54.4±0.5	57.5±0.0	55.2±1.2	55.1±0.6

Sum tetrapeptides	40.7±0.1	38.8±0.2	39.7±0.1	39.0±0.1	39.5±0.0	39.8±0.5
Sum pentapeptides	1.3±0.1	2.3±0.1	1.9±0.3	1.3±0.0	1.6±0.0	2.0±0.1
Degree of Crosslinkage[2]	39.8±0.1	38.1±0.4	38.9±0.3	38.2±0.3	38.2±0.2	39.2±0.6
% Peptides in Crosslinkage[3]	76.0±0.1	75.2±0.7	76.1±0.3	74.4±1.6	75.7±0.3	77.0±0.2
Sum amidated peptides	72.7±8.6	68.2±2.2	68.8±0.3	73.6±0.0	75.5±0.0	74.9±2.8

[1] Relative amounts of muropeptides isolated from several strains for this work. The muropeptides were assigned according to Atrih *et al.,* (1999)[1]. Values represent the mean ± variation from two independent PG preparations.
[2] Calculated according to Glauner *et al.,* (1988)[3]
[3] Calculated as 100% - % monomers.

References

1. Atrih, A., Bacher, G., Williamson, M. P. & Foster, S. J. Analysis of peptidoglycan structure from vegetative cells of *Bacillus subtilis* 168 and Role of PBP 5 in peptidoglycan maturation. *J. Bacteriol.* **181**, 3956–3966 (1999).

2. Bisicchia, P., Bui, N. K., Aldridge, C., Vollmer, W. & Devine, K. M. Acquisition of VanB-type vancomycin resistance by *Bacillus subtilis*: The impact on gene expression, cell wall composition and morphology. *Mol. Microbiol.* **81**, 157–178 (2011).

3. Glauner, B., Höltje, J. V. & Schwarz, U. The composition of the murein of *Escherichia coli*. *J. Biol. Chem.* **263**, 10088–95 (1988).

4. Meyer, H. *et al.* A time resolved metabolomics study: The influence of different carbon sources during growth and starvation of *Bacillus subtilis*. *Mol. Biosyst.* **10**, 1812–1823 (2014).

5. Dörries, K. & Lalk, M. Metabolic footprint analysis uncovers strain specific overflow metabolism and D-isoleucine production of *Staphylococcus aureus* COL and HG001. *PLoS One* **8**, e81500 (2013).

6. Weart, R. B. *et al.* A metabolic sensor governing cell size in bacteria. *Cell* **130**, 335–347 (2007).

7. Dominguez-Cuevas, P. *et al.* Differentiated roles for MreB-actin isologues and autolytic enzymes in *Bacillus subtilis* morphogenesis. *Mol Microbiol* **89**, 1084–1098 (2013).

8. Popham, D. L. & Setlow, P. Cloning, nucleotide sequence, and mutagenesis of the *Bacillus subtilis ponA* operon, which codes for penicillin-binding protein (PBP) 1 and a PBP- related factor. *J. Bacteriol.* **177**, 326–335 (1995).

9. Nicolas, P. *et al.* Condition-dependent transcriptome reveals high-level regulatory architecture in *Bacillus subtilis*. *Science* **335**, 1103–6 (2012).

10. Schirner, K., Marles-Wright, J., Lewis, R. J. & Errington, J. Distinct and essential morphogenic functions for wall- and lipo-teichoic acids in *Bacillus subtilis*. *EMBO J.* **28**, 830–842 (2009).

11. Yamamoto, H., Miyake, Y., Hisaoka, M., Kurosawa, S. & Sekiguchi, J. The major and minor wall teichoic acids prevent the sidewall localization of vegetative DL-endopeptidase LytF in *Bacillus subtilis*. *Mol. Microbiol.* **70**, 297–310 (2008).

12. Schirner, K. & Errington, J. Influence of heterologous MreB proteins on cell morphology of *Bacillus subtilis*. *Microbiology* **155**, 3611–3621 (2009).

13. Morimoto, T. *et al.* Six GTP-binding proteins of the Era/Obg family are essential for cell growth in *Bacillus subtilis*. *Microbiology* **148**, 3539–3552 (2002).

14. Veening, J. W., Kuipers, O. P., Brul, S., Hellingwerf, K. J. & Kort, R. Effects of phosphorelay perturbations on architecture, sporulation, and spore resistance in biofilms of *Bacillus subtilis*. *J. Bacteriol.* **188**, 3099–3109 (2006).
